# Melody: meta-analysis of microbiome association studies for discovering generalizable microbial signatures

**DOI:** 10.1186/s13059-025-03721-4

**Published:** 2025-08-18

**Authors:** Zhoujingpeng Wei, Guanhua Chen, Zheng-Zheng Tang

**Affiliations:** Department of Biostatistics and Medical Informatics, University of Wisconsin-Madison, Madison, WI 53715 USA

**Keywords:** Absolute abundance, Best subset selection, Compositional data, Meta-analysis, Quasi-multinomial model, Relative abundance, Signature harmonization, Summary statistics

## Abstract

Standard protocols for meta-analysis of association studies are inadequate for microbiome data due to their complex compositional structure, leading to inaccurate and unstable microbial signature selection. To address this issue, we introduce Melody, a framework that generates, harmonizes, and combines study-specific summary association statistics to powerfully and robustly identify microbial signatures in meta-analysis. Comprehensive and realistic simulations demonstrate that Melody substantially outperforms existing approaches in prioritizing true signatures. In the meta-analyses of five studies on colorectal cancer and eight studies on the gut metabolome, we showcase the superior stability, reliability, and predictive performance of Melody-identified signatures.

**Supplementary Information:**

The online version contains supplementary material available at 10.1186/s13059-025-03721-4.

## Background

Advanced sequencing technologies have enabled the study of microbial communities that colonize the human body in a culture-independent manner [1]. Microbiome association analysis is routinely performed to discover microbial signatures associated with covariates such as health outcomes, exposures, and molecular traits. The signature identified in one study is often hard to generalize to other cohorts and populations because of the small sample size in a single study. A natural way to increase sample size is to perform meta-analyses. Recent years have witnessed a surge in conducting meta-analyses of microbiome association studies [2–5]. Unfortunately, standard meta-analysis strategies commonly used in clinical and genomics studies fail to address the unique characteristics of microbiome data, undermining the ability of meta-analysis to identify generalizable microbial signatures.

Microbiome data generated from marker-gene or metagenomics sequencing contain sequencing read counts allocated to various microbial features, such as taxonomic and functional units [6]. The sequencing depth (the total number of reads obtained for a sample) is not correlated with the actual microbial load (the total amount of microorganisms or their DNA volumes) in the sample. Therefore, microbiome data are compositional data that represent the relative abundance (RA) of microbial features. These counts are distorted from the underlying absolute abundance (AA) per unit volume [7, 8]. Although techniques have been developed to recover AA data [9–11], the cost is prohibitive and the effectiveness is debatable because they cannot correct measurement biases arising from different efficiencies (e.g., DNA extraction efficiency, primer binding, and amplification efficiency) with which various features are measured [12]. The RA of all features would shift even if only one feature changes in AA. The small shift may be too subtle to affect the association analysis of a single study but would become more pronounced and impactful as the sample size and data heterogeneity accumulate in the meta-analysis.

Several compositionality-aware methods have been developed for microbiome association analysis [13–18]. These methods employ different strategies to address compositionality. Methods such as ANCOM-BC2 [17], LinDA [15], and radEmu [18] explicitly estimate and correct compositional bias to recover AA association coefficients. SRI [16] keeps the analysis on the relative scale but marginalizes over a prior for each sample’s unknown microbial load, so compositionality is treated as uncertainty rather than bias. DR [13] ranks features using log-ratio comparisons to a reference frame. While these methods are useful, they are not designed to estimate and harmonize AA association coefficients across studies for meta-analysis.

In addition to compositionality, other characteristics of microbiome data further compound the challenges of microbiome association meta-analyses. The sequencing depth strongly influences the sequencing read counts. Methods that fail to consider the variation in sequencing depth across samples can lead to a high number of false positives, particularly when the depths vary significantly between groups of comparison [19]. While rarefaction is commonly used to address uneven sequencing depth, it may reduce statistical power in certain analyses due to data loss introduced by subsampling [20, 21]. Moreover, for many rare features, insufficient sampling and the absence of features result in an excessive number of zero counts. Some features may be completely missing in a subset of studies in meta-analysis. While the imputation of zero to non-zero values is a widely adopted practice in processing microbiome data, consensus on the best method is lacking even for data from a single study, and it may introduce additional bias to the analysis [19, 22]. Filtering out rare features is another common practice under the belief that rare features are uninformative. However, recent research has noted an alarming phenomenon that analysis with different filtering criteria can result in identifying very different sets of microbial signatures [23, 24].

Pooling individual-level data and combining summary data are two types of meta-analysis strategies, each of which faces different challenges in microbiome association meta-analyses. Pooled-data meta-analysis requires careful harmonization of individual-level data that usually involves correcting batch effects in microbiome data across studies. The distribution of microbiome data can substantially differ among studies involving various age groups, genders, geographic regions, and measurement time points. Batch effects can also occur in sequencing experiments due to differences in reagents, equipment, protocols, and personnel. More importantly, batch effects are often entangled with association effects in microbiome studies [25–27]. Correcting batch effects while preserving the signal of interest is challenging due to the aforementioned characteristics of microbiome data. Prior studies in genomics have shown that batch effect correction may distort associations in pooled-data meta-analyses [28–30].

Summary-data meta-analysis circumvents the need for individual-level data harmonization, as summary statistics are generated for each study separately. This type of meta-analysis is preferable to pooled-data meta-analysis when there are differences in sample ascertainment and in data measurements and availability across studies. If the individual-level data is not shared, the logistical burden and privacy concerns can be significantly reduced, especially when the data contain sensitive information such as genetics or medical history. For these critical reasons, summary-data meta-analysis is widely adopted in many research fields, such as genetic association studies [31]. However, standard approaches for generating and combining summary statistics are inadequate for microbiome data due to their unique characteristics mentioned above.

We introduce a summary-data meta-analysis framework named Melody (meta-analysis for selecting microbial drivers in compositionality). Melody generates accurate and robust summary association statistics between the RA of microbial features and the covariate of interest by respecting the microbiome data characteristics. The procedure does not require microbiome data normalization, rarefaction, zero imputation, or batch effect correction across studies. By harmonizing and combining the summary statistics across studies, Melody identifies microbial signatures with their AA consistently associated with the covariate in all studies. Melody allows study-specific confounder adjustments, accommodates correlated samples, and is computationally efficient.

The recovery of AA associations in Melody relies on the assumption that the true signatures are “drivers,” defined as the minimal set of microbial features whose changes in AA are sufficient to explain the association signal observed at the RA level. The concept of driver signatures is illustrated in Additional file 1: Fig. S1. The figure demonstrates that the underlying AA association profile compatible with the same RA association profile (referred to as “putative AA association”) is not unique, leading to ambiguity in inferred signatures. Driver signatures are those features with nonzero effects in the sparsest putative AA association profile. The figure also shows that driver signatures may or may not overlap with features exhibiting nonzero RA associations. In subsequent sections, we show that focusing on identifying drivers is crucial for harmonizing RA summary statistics across studies and for discovering stable, generalizable microbial signatures in meta-analyses.

## Results

### Melody overview

**Fig. 1:**
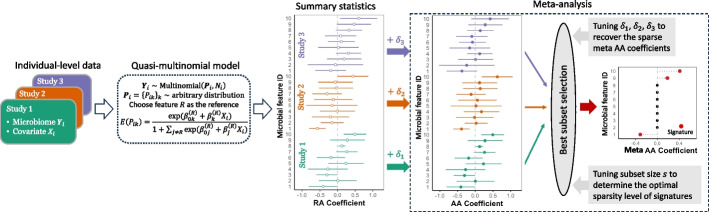
Schematic overview of Melody. The Melody framework begins by generating summary statistics for each study using the quasi-multinomial model. In the model, the vector of true proportions ${\textbf {P}}_i$ is allowed to follow an arbitrary distribution, and only its mean is modeled to link with the covariate of interest $X_i$. The $\beta ^{(R)}_k$ is the relative abundance (RA) association coefficient of feature *k* given the reference feature *R*. The panel for summary statistics displays the estimates of the RA association coefficient (open dots) and the estimates plus or minus standard errors (horizontal lines). Next, the RA coefficient estimates in each study $\ell$ are shifted by $\delta _\ell$. These $\delta _\ell$ values are jointly tuned with the sparsity level hyperparameter *s* for the best subset selection. In this illustration, the optimal value for $\delta _1$ is close to zero, for $\delta _2$ is positive, and for $\delta _3$ is negative. As a result, the RA coefficients in Study 2 are shifted to the right, and those in Study 3 are shifted to the left to obtain the corresponding estimates of study-specific absolute abundance (AA) association coefficients (solid dots). These study-specific coefficients are then combined to estimate meta AA association coefficients. The optimal $\delta _\ell$ values ensure that the RA coefficients are shifted appropriately to harmonize signals across studies and recover sparse meta AA associations

Melody is described in the “Methods” section; its technical details are provided in the Additional file 1: Supplementary Note A, and its schematic overview is shown in Fig. 1. For each study, we link the microbiome count data with the covariate of interest using a quasi-multinomial regression model and obtain summary statistics consisting of the estimates of the RA association coefficients and their variances. The RA association coefficients are estimated based on the scaled score statistics, which are numerically stable, statistically accurate, and computationally efficient [32, 33]. The quasi-multinomial framework accommodates overdispersion in microbiome count data and mitigates the risk of inaccurate inference associated with the strict multinomial assumption. The model easily accommodates confounder adjustments and naturally handles correlated samples (e.g., from longitudinal studies). The RA association coefficients in the model are formulated at the log-ratio scale, with a specified feature as the reference. The RA association coefficients are expressed as the shifted AA association coefficients by a constant (“Methods” section).

Next, we combine the RA summary statistics across studies to estimate the sparse meta AA association coefficients. This meta-analysis is framed as a best subset selection problem [34, 35], where we employ a cardinality constraint with the subset size hyperparameter *s* to encourage the sparsity of meta AA association coefficients. To recover meta coefficients at the AA level using summary statistics at the RA level, we introduce a hyperparameter $\delta _\ell$ in each study $\ell$ to shift the RA association coefficient estimates, producing different versions of putative AA association coefficients by varying $\delta _\ell$. These study-specific AA coefficients are then combined to estimate meta AA coefficients. The hyperparameters *s* and $\delta _\ell$’s are jointly tuned using the Bayesian information criterion (BIC) to balance the trade-off between the model fit and sparsity. As illustrated in Fig. 1, the optimal $\delta _\ell$ values shift the RA coefficients by an appropriate amount to identify a version of putative AA association coefficients that are sparse and consistent across studies. The Melody-identified driver signatures are microbial features with non-zero estimates of meta AA association coefficients under the optimal hyperparameters.

It is important to note that while we need to select a reference feature for generating summary statistics, the signature selection is not sensitive to the choice of the reference. We can even choose different references for different studies (see the sensitivity analysis in the real data application). This is because the reference effects across studies are offset by $\delta _\ell$’s in the process of estimating the sparse meta AA association coefficients.

### Simulations

In the simulations, we compared Melody with the MMUPHin summary-data meta-analysis method and several pooled-data meta-analysis approaches for selecting microbial signatures with differential abundance across two groups of interest. MMUPHin [5] is an R package that includes a batch-effect correction algorithm and a summary-data meta-analysis method. The meta-analysis first generates summary statistics using MaAsLin2 [36] within individual studies and then combines these summary statistics using standard meta-analysis methods [37] for association testing. In the pooled-data meta-analysis, the pooled data were analyzed using representative microbiome association tests ALDEx2 [38], ANCOM-BC2 [17], and the blocked Wilcoxon test (BW) [3], as well as a penalized regression method CLR-LASSO [39]. In ALDEx2 and ANCOM-BC2, we adjusted for studies as confounders. In BW, we rarefied count data within each study and performed the blocked Wilcoxon rank-sum test with studies specified as blocks. In CLR-LASSO, we applied LASSO logistic regression [40] using centered log-ratio (CLR) transformed microbiome data as predictors. Melody was applied to the original data, and the compared methods were applied to both the original data and study-effect corrected data using the MMUPHin batch-effect correction algorithm. We compared the performance using the area under the precision-recall curve (AUPRC) for signature selection. Specifically, the precision-recall curves of the test-based methods (MMUPHin, BW, ALDEx2, ANCOM-BC2) were constructed by changing the *p*-value cutoff, and those of the penalized methods (Melody and CLR-LASSO) were constructed by varying the hyperparameter that controls the signature sparsity level.

**Fig. 2:**
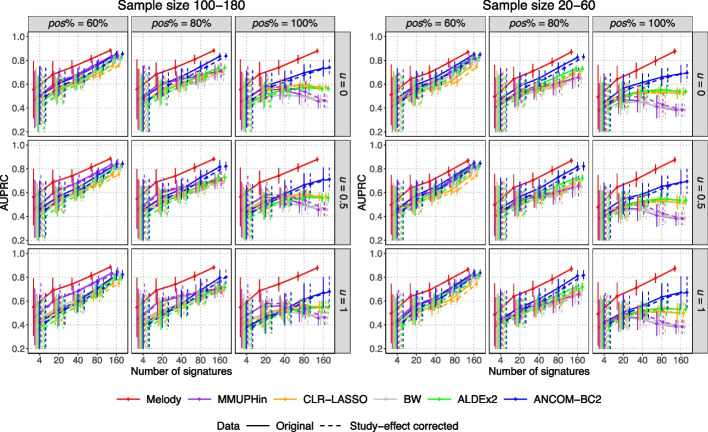
Precision-recall evaluation of different meta-analysis signature selection approaches in simulated data with independent samples from five studies. Melody was applied to the original data of the five studies, and the other methods were applied to both the original data and study-effect corrected data using the MMUPHin batch-effect correction algorithm. The *x*-axis represents the number of signatures. The left and right sections show the results under different sample size settings (the sample size range for the five studies is 100–180 or 20–60). Within each section, the columns of the plots represent various degrees of sign imbalance among signatures ($pos\%$ denotes the percentage of signatures with positive effects); the rows of the plots represent various degrees of sequencing depth unevenness between the two groups of interest (*u* denotes the sequencing depth relative change ratio for the group with higher sequencing depth, and 0 indicates no unevenness). Each panel displays the mean area under the precision-recall curve (AUPRC) with ± standard errors (indicated by error bars) based on 100 simulation replicates

We simulated microbiome data for five studies that resemble the structure of five real microbiome datasets [3] (Additional file 1: Fig. S2). The detailed simulation strategy is described in the “Methods” section. In brief, we consider a wide range of settings by varying the study sample size, the number of signatures, the degree of sign imbalance among signatures, and the degree of sequencing depth unevenness between the two groups of interest. Melody produces the highest AUPRC values in all settings and is robust with varying degrees of sign imbalance and sequencing depth unevenness (Fig. 2). The AUPRC of ANCOM-BC2 reduces substantially as the degrees of the two factors increase. The AUPRC of MMUPHin, CLR-LASSO, BW, and ALDEx2 reduce significantly as the degree of sign imbalance increases. When using the study-effect corrected data, the AUPRC of MMUPHin slightly increases, while the pooled-data meta-analysis methods show minimal change. We performed another set of simulations for the meta-analysis of studies with correlated samples and compared Melody with MMUPHin and ANCOM-BC2, which can handle correlated samples. The patterns of the results remain the same (Additional file 1: Fig. S3).

To strengthen our benchmarking, we ran an additional suite of simulation studies based on a different data-generation scheme (Additional file 1: Supplementary Note B). We first simulated AA data, introduced AA-level differential abundance, and then added study-specific, feature-level measurement biases to mimic technical batch effects. We further varied the batch-effect magnitude, number of studies, and association strength. Across all of these scenarios, Melody consistently outperforms competing methods (Additional file 1: Fig. S4).

### Meta-analysis of microbiome association studies for colorectal cancer

We conducted a meta-analysis of five metagenomics studies [3, 41–44] for colorectal cancer (CRC) to identify microbial signatures associated with CRC. The data contain 849 gut bacterial species measured on 574 fecal samples across the five studies (Additional file 1: Table S1). Microbial communities display a pattern of cross-study heterogeneity (Additional file 1: Fig. S2). The sequencing depth between CRC cases and controls is significantly different in some studies (Additional file 1: Fig. S5). Our main analysis focused on 401 microbial species with at least $20\%$ prevalence across all samples in the five studies. We applied the methods that were compared in the simulations to analyze the data. For the test-based methods, the signatures were selected using the Benjamini-Hochberg (BH) procedure [45] at the false discovery rate (FDR) level of 0.05. For CLR-LASSO, the signatures were selected based on the best LASSO model. The model tuning was performed via five-fold cross-validation using the default settings of the cv.glmnet function from the glmnet R package (v4.1-8), which selects the hyperparameter by minimizing the cross-validated binomial deviance.

**Fig. 3:**
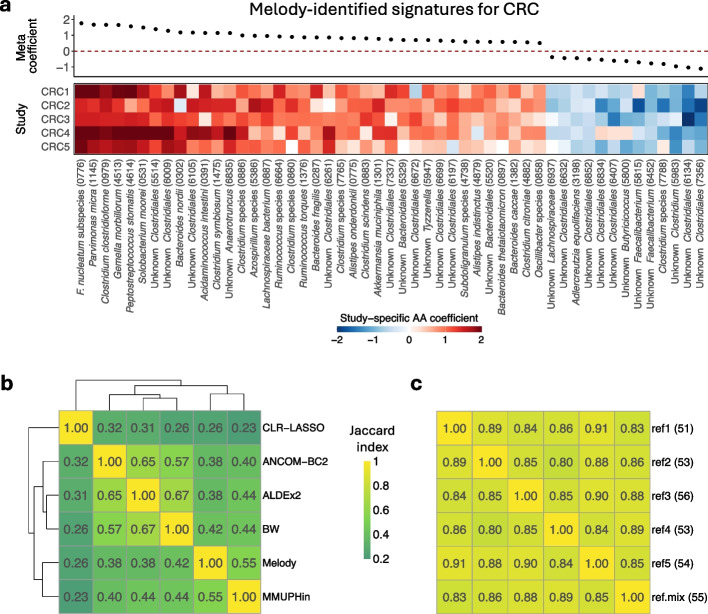
Meta-analysis results from five metagenomics studies for identifying signatures for colorectal cancer (CRC). **a** Estimates of the absolute-abundance (AA) association coefficient for the 51 signatures identified by Melody. The scatter plot displays the estimates of the meta coefficients, and the heatmap displays the estimates of the study-specific coefficients. **b** Heatmap of the Jaccard index measuring the overlap of the top 51 selected species across different methods. The dendrogram is constructed using hierarchical clustering of the Jaccard index matrix. Results of all methods were generated on the original data. **c** Heatmap of the Jaccard index measuring the overlap of the Melody-identified signatures across different reference configurations. The ref1 represents the scenario where we use species *Coprococcus catus* as the reference in all studies. The ref2-ref5 represent scenarios where we replace ref1 with a different reference and use the reference in all studies. The ref.mix represents the scenario where we use different references for different studies (ref1-ref5 for CRC1-CRC5). The number in parentheses is the count of the identified signatures

The meta-analysis results of all methods are presented in Additional file 2: Table S2. Melody identified 51 signatures for CRC (Fig. 3a), with the top two signatures *Fusobacterium nucleatum* and *Parvimonas micra* being the well-known species promoting CRC [46, 47]. Using original data, MMUPHin, CLR-LASSO, BW, ALDEx2, and ANCOM-BC2 selected 84, 94, 110, 44, and 72 signatures, respectively. Signatures identified on the study-effect corrected data are similar to those identified on the original data. The top-selected species and their rankings vary markedly across methods (Fig. 3b, Additional file 1: Figs. S6 and S7).

We used species *Coprococcus catus* as the reference when generating Melody summary statistics for individual studies. We also chose other references and evaluated the sensitivity of the Melody result. Additionally, we used different references for different studies and repeated the analysis. The Melody signatures are highly consistent across these reference configurations (Fig. 3c). Although the RA association coefficient estimates show noticeable differences across various choices of reference, the Melody-recovered AA association coefficients are almost invariant (Additional file 1: Fig. S8). The AA coefficients also show better harmonization with consistent association directions across studies than the RA coefficients.

**Fig. 4:**
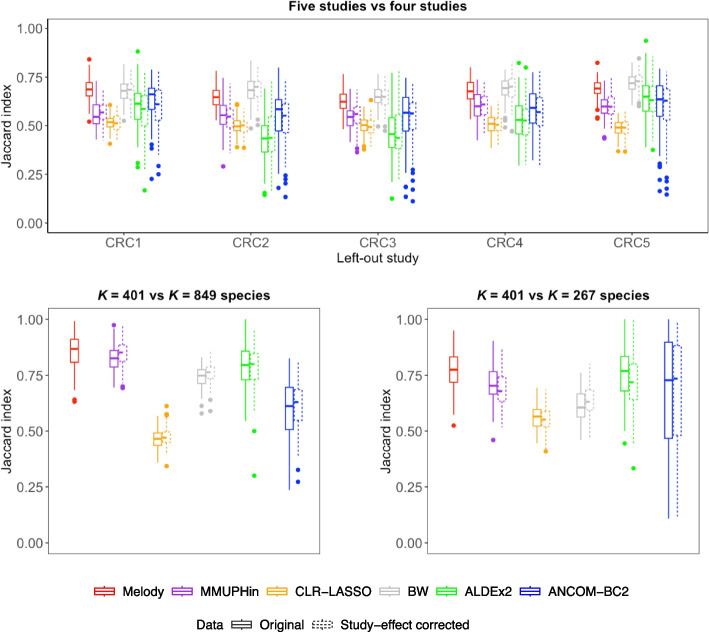
Comparison of the stability of signature selection of different methods when performing two sets of meta-analyses based on metagenomics studies for colorectal cancer. Melody was applied to the original data, and the other methods were applied to both the original data and study-effect corrected data. The Jaccard index measures the overlap of the identified signatures from two sets of meta-analyses. Upper panel: Analysis 1 is based on data from all five studies, and Analysis 2 is based on data with one study left out. Lower left panel: Analysis 1 includes $K = 401$ species, and Analysis 2 includes all $K = 849$ species. Lower right panel: Analysis 1 includes $K = 401$ species, and Analysis 2 includes $K = 267$ species under order *Clostridiales*. Box plots were derived from 100 bootstrap samples of the complete data from the five studies. Each box indicates the median (center line), the first and third quartiles (box edges), whiskers extending to 1.5 times the interquartile range, and individual outliers (dots)

We evaluated the stability of signature selection from three perspectives. (1) Is the selection stable under different subsets of the samples? We conducted a sensitivity analysis by removing one study at a time from the meta-analysis. Specifically, we excluded all samples from a single study, identified signatures based on the remaining four studies, and evaluated the overlap of the signatures with those identified using the full dataset comprising all five studies. (2) Is the selection stable after applying different prevalence filters? Our main analysis was on $K=401$ species after applying the 20% prevalence filter. We also identified signatures based on all $K=849$ species, kept the subset of the signatures within the $K=401$ species list, and evaluated the overlap of the subset with those selected in the main analysis. (3) Is the selection stable if analyzing a subcomposition? We identified signatures based on a subcomposition with $K=267$ species under order *Clostridiales*. We evaluated the overlap of the identified signatures with the subset of main-analysis-identified signatures within the $K=267$ species list. We performed 100 bootstrap resamples of the combined data (i.e., bootstrap resampling by randomly drawing, with replacement, a sample of the same size from each study, and then combined the resampled datasets across studies), repeated these stability evaluations on the bootstrap samples, and reported the Jaccard index to measure set overlap (Fig. 4). When one study is excluded from the meta-analysis, Melody exhibits higher stability compared to MMUPHin, CLR-Lasso, ALDEx2, and ANCOM-BC2 (Wilcoxon rank-sum test FDR-adjusted $P < 0.001$); BW shows comparable stability to Melody when CRC1 or CRC4 is excluded, and has slightly higher stability when CRC2, CRC3, or CRC5 are excluded (Wilcoxon rank-sum test FDR-adjusted $P < 0.1$). When the set of microbial features is larger ($K=849$), the stability of Melody is much higher than that of ALDEx2, ANCOM-BC2, BW, and CLR-Lasso (Wilcoxon rank-sum test FDR-adjusted $P < 10^{-6}$). When the set of microbial features is smaller ($K=267$), the stability of Melody is much higher than that of BW, CLR-Lasso, and MMUPHin (Wilcoxon rank-sum test FDR-adjusted $P < 10^{-6}$).

**Fig. 5:**
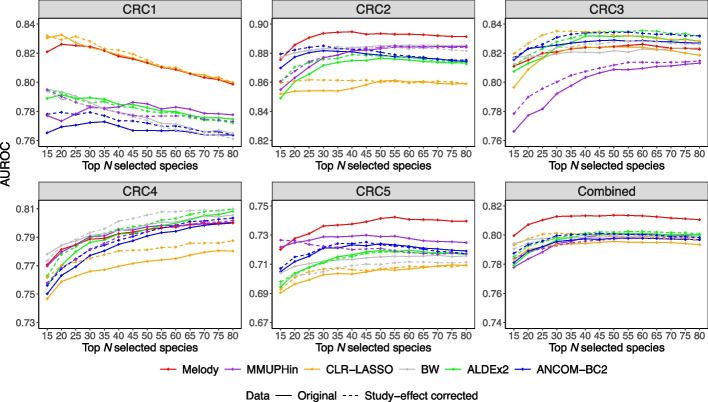
Comparison of colorectal cancer (CRC) prediction using the top species identified by different meta-analysis methods. One study was held out each time for prediction evaluation, and samples from the remaining studies were randomly split into a feature selection set and a prediction model training set. Meta-analysis methods were applied to the feature selection set to identify the top *N* ($N=15, \ldots , 80$) CRC-associated species. Melody was applied to the original data, and the other methods were applied to both the original data and study-effect corrected data. A random forest model for CRC prediction was trained using the sample proportions of the top species in the training set, and the best model was evaluated on the hold-out study based on the area under the receiver operating characteristic (AUROC). The procedure was repeated 100 times with different random splits of the samples. In the first five panels, the average AUROC is provided in each panel, with the hold-out study ID listed at the top. The “Combined” panel displays the average AUROC across all hold-out studies

In addition, we evaluated the out-of-sample prediction power of the top-selected species using the leave-one-study-out (LOSO) strategy. We held out one study for prediction evaluation and randomly split samples from the remaining four studies into a feature selection set and a prediction model training set at a sample size ratio of 3:1. We performed the meta-analysis on the feature selection set to pick the top *N* CRC-associated species. We then used the sample proportions of these species in the training set to train a random forest model for CRC prediction. Finally, we calculated the area under the receiver operating characteristic (AUROC) of the best random forest model on the hold-out study. We repeated the procedure 100 times with different random splits of the samples and reported the average AUROC (Fig. 5). The Melody-identified top species have substantially higher AUROC than those identified by the test-based methods in CRC1, CRC2, and CRC5 (Wilcoxon rank-sum test FDR-adjusted $P<10^{-5}$), and are comparable to the highest AUROC in other studies. The top species identified by CLR-LASSO performed well in CRC1 but much worse in CRC2, CRC4, and CRC5. Overall, the group of species prioritized by Melody has the highest average AUROC in CRC prediction (Wilcoxon rank-sum test FDR-adjusted $P<10^{-10}$), whereas the species prioritized by other methods yielded similar results (Combined panel in Fig. 5).

### Meta-analysis of microbiome-metabolome association studies

We conducted another meta-analysis of eight microbiome-metabolome association studies [48–55] to identify microbial signatures associated with human gut metabolites. Most of these studies are case-control studies with one group of individuals with a specific medical condition and the other group of healthy control individuals (Additional file 1: Table S3). The raw data contain 12195 gut bacterial genera and 1151 HMDB-annotated [56] metabolites measured on 2127 fecal samples across the eight studies. We focused on metabolites and genera with at least $10\%$ prevalence within individual studies. Most metabolites are available in only a subset of studies (Additional file 1: Fig. S9). We considered metabolites that are present in at least two studies. The final datasets for analysis contain 450 metabolites and 101 genera, resulting in 43722 metabolite-genus pairs. There is notable heterogeneity among studies in study designs, microbiome sequencing methods, and metabolite measurements (Additional file 1: Table S3). In particular, metabolite levels cannot be directly compared between studies due to differences between metabolomics platforms. Short-chain fatty acids, for example, are primarily detected using gas chromatography-mass spectrometry (GC-MS) and are rarely detected by liquid chromatography-mass spectrometry (LC-MS) due to their poor ionization efficiency. Therefore, the pooled-data meta-analysis is unsuitable and we performed Melody and MMUPHin summary-data meta-analyses on the original data. In both methods, we analyzed each metabolite variable as a covariate of interest while adjusting the disease status as a confounder when generating summary statistics for individual studies. For MMUPHin, the signatures for each metabolite were selected using the BH procedure at the FDR level of 0.05.

**Fig. 6:**
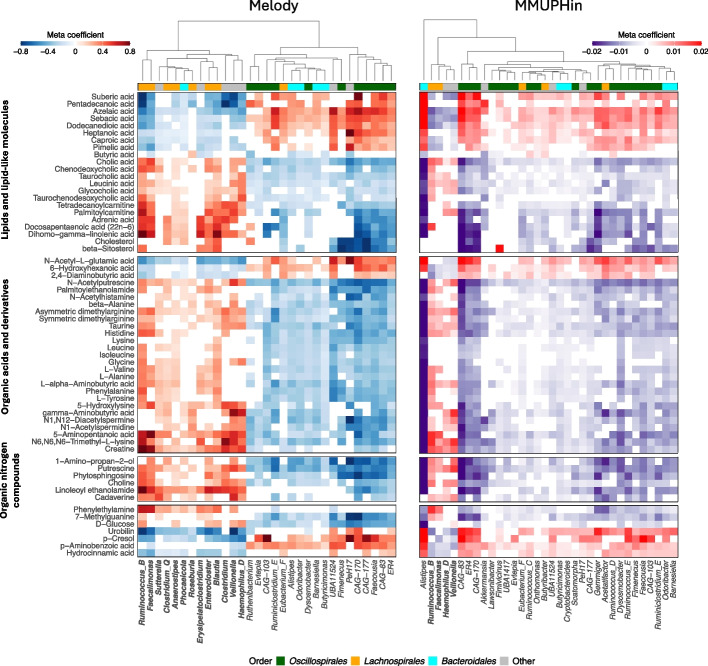
Heatmaps of meta association coefficient estimates for the Melody and MMUPHin identified metabolite-genus pairs. The coefficient is shown as zero if the method does not select the metabolite-genus pair. The rows of both heatmaps are for the same set of top metabolites that are associated with at least 30 genera in Melody results. Most of the metabolites are grouped into three HMDB superclasses (names shown on the leftmost), while the remaining metabolites are placed at the bottom rows. The columns of the two heatmaps show genera associated with at least 70 metabolites, one from Melody results and the other from MMUPHin results. The names of the genera that have disproportionately positive associations with the top metabolites are in bold. Bacterial orders are summarized by the color code. Rows within each superclass and columns are clustered by Euclidean distance

Melody completed the analysis within 20 min, while MMUPHin took 25 min on a laptop (Apple M1 Pro 3.2GHz processor). Melody identified 5847 metabolite-genus pairs, among which 2499 have positive and 3348 have negative associations (Additional file 2: Table S4). MMUPHin identified 5921 pairs, among which 2065 have positive and 3856 have negative associations (Additional file 2: Table S5). Figure 6 displays the meta association coefficient estimates of the two methods for the same set of top metabolites, which are associated with a large number of genera. Melody identifies a greater number of genera that are positively associated with the majority of top metabolites, compared to MMUPHin (Fig. 6). These genera are associated with much fewer metabolites in MMUPHin results (Additional file 1: Fig. S10b). Many of them, such as *Anaerostipes*, *Blautia*, and *Roseburia*, are known to play essential roles in key metabolic processes, including the fermentation of dietary fibers and diverse carbohydrates [57, 58], and are closely associated with the production of short-chain fatty acids, bile acids, and amino acid derivatives. These genera have also been linked with multiple human complex diseases such as metabolic syndrome, inflammatory bowel disease, and obesity [59–61]. Besides the direct engagement of the genus in metabolite production, consumption, or degradation, correlations between metabolites and genera can also result from indirect associations, such as interactions between gut bacteria or co-abundant metabolites.

We further examined metabolites for which the results of the two methods were strikingly different. Additional file 1: Fig. S11 highlights one such metabolite, 4-trimethylammoniobutanoate (also known as gamma-butyrobetaine), a key gut microbial intermediate in the metabolism of L-carnitine to trimethylamine N-oxide (TMAO) with demonstrated proatherogenic effects in preclinical models [62]. Melody identified 13 positive-associated genera and 10 negative-associated genera, whereas MMUPHin identified 7 positive-associated genera and 21 negative-associated genera. For each method, we removed 5 genera with the highest positive meta association identified by the method and reran the meta-analysis. The Melody signature selection is stable after the genus removal. MMUPHin identified many new positive-associated genera, and the set of negative-associated genera also changed substantially. This is a global phenomenon, not limited to a few metabolites. We performed the same evaluation for all 106 metabolites with at least 5 positive-associated genera detected by Melody and MMUPHin. The overlap of the signatures before and after genus removal is much higher in Melody than in MMUPHin for most of these metabolites (Additional file 1: Fig. S12). The stability of the Melody results shown in this experiment is mainly attributed to its ability to capture the fluid association signals in the compositional data. This evaluation underscores the importance of addressing compositionality to ensure the stability and reliability of results in microbiome association meta-analyses.

## Discussion

We have developed Melody to address major challenges in the meta-analysis of microbiome association studies. Melody’s key innovations are (a) generating accurate and robust summary statistics by accounting for the unique characteristics of microbiome data; (b) recovering meta association coefficients at the absolute abundance level and identifying driver signatures consistent across studies. Melody has a unique combination of features that make it desirable for practical use: (1) no need to share individual-level data or to correct study effects; (2) no need to perform data normalization, rarefaction, or zero imputation; (3) no need to assume that the same set of microbial features is measured and analyzed across studies; (4) allows for different designs across studies (e.g., some studies are cross-sectional, and some are longitudinal); (5) easily adjusts for confounders and allows different sets of confounders available across studies; (6) fast computation for large-scale association scans.

Melody tackles compositionality in a novel way. It corrects study-specific compositional biases by jointly tuning the per-study scaling factors $\delta _\ell$ and the signature size *s* within a best subset selection framework, yielding consistent AA estimates. Joint tuning is critical and simply fixing $\delta _\ell$ to the median of the RA coefficient estimates fails to achieve comparable performance (Additional file 1: Fig. S13). However, Melody’s effectiveness rests on a sparsity assumption. In simulations where more than half of the features are driver signatures, the $\delta _\ell$ estimates degrade and Melody does not outperform the compared methods (Additional file 1: Fig. S14). Note, however, that observing RA shifts in many features does not automatically imply a dense signal; large effects from a small set of drivers can induce widespread apparent shifts because of compositional constraints.

As demonstrated in the real data application, the set of Melody-identified signatures is not sensitive to the prevalence filter. However, like any method for high-dimensional feature selection, including lots of noise features could adversely impact the selection accuracy. Therefore, we still suggest applying a prevalence filter before data analysis if investigators are not interested in detecting very rare features. The filter can be applied to pooled samples of all studies (as in the meta-analysis for CRC) or samples within each study in case a feature may be more prevalent in some studies and worth keeping (as in the meta-analysis for metabolome). Melody naturally accommodates different sets of features across studies in meta-analysis.

This article focuses on identifying microbial signatures that are shared across studies, as these are more likely to be generalizable to broader populations. It can also be of interest to identify microbial signatures with heterogeneous associations across populations (e.g., microbial features associated with the covariate in females but not in males) [63]. Melody can be extended to detect such heterogeneous associations by adapting random-effects meta-analysis and meta-regression techniques [64].

## Conclusions

Melody is a new meta-analysis framework that enables the identification of true microbial signatures with absolute-abundance level associations using microbiome relative-abundance data. Compared to existing meta-analysis approaches, Melody substantially improves cross-study signal harmonization and enhances signature detection accuracy and stability. With the ever-increasing number of microbiome datasets, we anticipate that Melody will facilitate the accumulation of sufficiently large sample sizes to accelerate the reliable discovery of generalizable microbiome signatures. As a general meta-analysis framework, Melody can be applied to the integration of association studies involving high-dimensional compositional omics data. We have provided an efficient R package (miMeta) for the broad utility of Melody.

## Methods

### Melody framework

*Generating summary statistics for individual studies.* The first step of Melody is to obtain summary association statistics between microbial composition and the covariate of interest in each participating study. Suppose we have *n* independent microbiome samples with *K* features in a given study. For sample *i*, let $X_i$ denote the covariate, $N_i$ denote the sequencing depth of the microbiome data, $Y_{ik}$ denote the number of sequencing reads assigned to feature *k* (i.e., microbiome count data), and $W_{ik}$ denote the unobserved AA of feature *k*. It is natural to model the mean of AA as

$$\begin{aligned} E(W_{ik}) = \exp (\beta _{0k} + \beta _k X_{i}),\ k = 1,\ldots ,K, \end{aligned}$$

where $\beta _{0k}$ is the intercept and $\beta _{k}$ represents the AA association coefficient for feature *k*. The relation between the RA mean and the AA mean can be expressed as

1 $$\begin{aligned} E(Y_{ik}) = c_i b_k E(W_{ik}) = c_i b_k \exp (\beta _{0k} + \beta _k X_{i} ), ~ k = 1,\ldots ,K, \end{aligned}$$

where $c_i$ is the sample-level multiplicative factor that absorbs quantities such as the sequencing depth and the microbial load in a unit volume for a sample, and $b_k$ is the feature-level multiplicative factor that absorbs the measurement biases arising from different efficiencies (e.g., DNA extraction efficiency, primer binding, and amplification efficiency) with which various features are measured [12].

The AA coefficients are not identifiable in the model (1). Therefore, we choose a feature *R* as the reference and construct the proportion mean parameter as

2 $$\begin{aligned} p_{ik} = \frac{E(Y_{ik})}{\sum \nolimits _{j=1}^K E(Y_{ij}) } = \frac{\exp (\beta ^{(R)}_{0k} + \beta ^{(R)}_k X_{i} )}{1+\sum \nolimits _{j \in \backslash R } \exp (\beta ^{(R)}_{0j} + \beta ^{(R)}_{j} X_{i})}, \end{aligned}$$

3 $$\begin{aligned} \beta ^{(R)}_{0k} = \log (b_k)+\beta _{0k} - \log (b_R)-\beta _{0R} \text { and } \beta ^{(R)}_k = \beta _k - \beta _R, ~ k \in \backslash R, \end{aligned}$$

where $\backslash R$ is the index set of non-reference features, and $\beta ^{(R)}_{0k}$’s and $\beta ^{(R)}_k$’s are the RA intercept and association coefficients, respectively. Note that the RA association coefficients of the non-reference features $\beta ^{(R)}_k$’s are simply shifted AA association coefficients $\beta _k$’s by a constant $\beta _R$.

The model can be extended to incorporate confounding variables. Let ${\textbf {X}}_i$ denote the design matrix that includes the intercept and all the explanatory variables and $\varvec{\theta }^{(R)} = (\varvec{\beta }^{(R)}, \varvec{\gamma }^{(R)})$ be the collection of all coefficients, in which $\varvec{\beta }^{(R)}$ contain the RA association coefficients of interest and $\varvec{\gamma }^{(R)}$ contain the nuisance coefficients.

We propose a quasi-multinomial model to estimate the coefficients. Specifically, given the true proportions of various features $(P_{i1}, \ldots , P_{iK})$, the counts $(Y_{i1}, \ldots , Y_{iK})$ naturally arise from multinomial sampling with parameters $(P_{i1}, \ldots , P_{iK})$ and size $N_i$. The multinomial distribution models microbial features competing for the sequencing reads bounded by the sequencing depth $N_i$ (i.e., allocating more reads to one feature means that fewer reads are available to assign to other features). The multinomial layer is particularly important for properly accounting for the effect of uneven sequencing depth on the variability of the counts given the true proportions. To address the over-dispersion, zero-inflation, and complex structure of the microbiome data, an additional layer of randomness is needed for $(P_{i1}, \ldots , P_{iK})$. We do not specify a particular distribution for this layer but only assume the proportion mean model $E(P_{ik})=p_{ik}$ specified in (2). To estimate the coefficients, we propose the following quasi-score function

$$\begin{aligned} \varvec{S}(\varvec{\theta }^{(R)}) = \sum \limits _{i=1}^n (\varvec{Y}_{i} - N_{i} {\textbf {p}}_{i}) \otimes {\textbf {X}}_i, \end{aligned}$$

where $\otimes$ represents the Kronecker product, $\varvec{Y}_i = \left\{ Y_{ik}\right\} _{k\in \backslash R}$, and ${\textbf {p}}_{i} = \left\{ p_{ik}\right\} _{k\in \backslash R}$. The parameters can be consistently estimated by solving the estimating equation $\varvec{S}(\varvec{\theta }^{(R)}) = \varvec{0}$. This is equivalent to finding the maximum likelihood estimator from multinomial logistic regression because the quasi-score function has the same form as the score function of multinomial regression. To prevent the potential estimation bias due to the small sample size and the presence of rare features, we adopted the mean bias reduction procedure in estimating the parameters of the multinomial regression [65].

Let $\varvec{S}_{\varvec{\beta }}$ and $\varvec{S}_{\varvec{\gamma }}$ denote the components in $\varvec{S}$ corresponding to $\varvec{\beta }^{(R)}$ and $\varvec{\gamma }^{(R)}$, respectively. Score statistics have been shown to be more accurate and numerically stable than Wald statistics in genetic association studies [32]. In light of this, we propose estimating $\varvec{\beta }^{(R)}$ based on the scaled score statistics

$$\begin{aligned} \widehat{\varvec{\beta }}^{(R)} = \varvec{\mathcal {I}}_{\varvec{\beta }}^{-1} (\widetilde{\varvec{\theta }}^{(R)}) \varvec{S}_{\varvec{\beta }}(\widetilde{\varvec{\theta }}^{(R)}), \end{aligned}$$

where $\widetilde{\varvec{\theta }}^{(R)} = (\varvec{0}, \widetilde{\varvec{\gamma }}^{(R)})$ and $\widetilde{\varvec{\gamma }}^{(R)}$ is the estimate of ${\varvec{\gamma }}^{(R)}$ under the reduced model without the covariate of interest in ${\textbf {X}}_i$, $\varvec{\mathcal {I}}_{\varvec{\beta }} = \varvec{\mathcal {I}}_{\varvec{\beta }\varvec{\beta }} - \varvec{\mathcal {I}}_{\varvec{\beta }\varvec{\gamma }} \varvec{\mathcal {I}}^{-1}_{\varvec{\gamma }\varvec{\gamma }} \varvec{\mathcal {I}}_{\varvec{\gamma }\varvec{\beta }}$, and $$\left[ \begin{array}{cc} \varvec{\mathcal {I}}_{\varvec{\beta }\varvec{\beta }} & \varvec{\mathcal {I}}_{\varvec{\beta }\varvec{\gamma }}\\ \varvec{\mathcal {I}}_{\varvec{\gamma }\varvec{\beta }} & \varvec{\mathcal {I}}_{\varvec{\gamma }\varvec{\gamma }} \end{array}\right]$$ is the partition of the quasi-information matrix $-\frac{\partial \varvec{S}(\varvec{\theta }^{(R)})}{\partial \varvec{\theta }^{(R)}}$ for $\varvec{\beta }^{(R)}$ and $\varvec{\gamma }^{(R)}$.

The sandwich estimator of the covariance of $\widehat{\varvec{\beta }}^{(R)}$ can be written as

$$\begin{aligned} {\varvec{V}}^{(R)} = \varvec{\mathcal {I}}_{\varvec{\beta }}^{-1}(\widetilde{\varvec{\theta }}^{(R)}) \left( \sum \limits _{i=1}^n {\varvec{U}}_i(\widetilde{\varvec{\theta }}^{(R)}){\varvec{U}}_i^{\text {T}}(\widetilde{\varvec{\theta }}^{(R)})\right) \varvec{\mathcal {I}}_{\varvec{\beta }}^{-1}(\widetilde{\varvec{\theta }}^{(R)}), \end{aligned}$$

where ${\varvec{U}}_i = \varvec{S}_{\varvec{\beta },i} - \varvec{\mathcal {I}}_{\varvec{\beta }\varvec{\gamma }} \varvec{\mathcal {I}}^{-1}_{\varvec{\gamma }\varvec{\gamma }} \varvec{S}_{\varvec{\gamma },i}$, $\varvec{S}_{\varvec{\beta },i}$ and $\varvec{S}_{\gamma ,i}$ are the *i*th summand of $\varvec{S}_{\varvec{\beta }}$ and $\varvec{S}_{\varvec{\gamma }}$ respectively. The correlations cannot be accurately estimated when the number of features is close to or larger than the sample size. Therefore, we use the diagonal matrix (with variances on the diagonal elements) extracted from ${\varvec{V}}^{(R)}$ in the meta-analysis. This procedure of generating summary statistics can be modified for studies with correlated samples (e.g., from longitudinal studies), and the details are provided in the Additional file 1: Supplementary Note A.

*Harmonizing and combining summary statistics across studies to identify signatures.* Suppose we want to meta-analyze *L* independent microbiome association studies and identify microbial signatures shared by the studies. Let $\varvec{\beta }_{\bullet \ell }$ and $\varvec{\beta }^{(R)}_{\bullet \ell }$ respectively denote the AA and RA association coefficients for study $\ell$. The length of these coefficients could differ among studies because a feature may not be observed in all studies. Let $\mathcal {T}_\ell$ be the index set of non-reference features observed in study $\ell$. For each study $\ell$, we obtain RA association coefficient estimates denoted by $\widehat{\varvec{\beta }}^{(R)}_ {\bullet \ell } = \{ \widehat{\beta }^{(R)}_{k\ell } \}_{k\in \mathcal {T}_\ell }$ and the diagonal matrix ${\varvec{V}}^{(R)}_\ell$ in which the diagonal elements are the variance of $\widehat{\varvec{\beta }}^{(R)}_{\bullet \ell }$. In the meta-analysis, we are interested in estimating the meta AA association coefficients that are common across all study-specific AA coefficients $\varvec{\beta }_{\bullet \ell }$ ($\ell =1,\ldots , L$). Let $\mathcal {T}_0$ denote the union set of all $\mathcal {T}_\ell$’s, $K_0$ denote the size of $\mathcal {T}_0$, and $\varvec{\mu } = \{\mu _k\}_{k\in \mathcal {T}_0}$ be the collection of the meta AA association coefficients for all the non-reference features appear in at least one study. We propose to estimate $\varvec{\mu }$ by minimizing the objective function

4 $$\begin{aligned} \mathcal {L}(\varvec{\mu }) ={n_0^{-1}}\sum \limits _{\ell =1}^L (\widehat{\varvec{\beta }}^{(R)}_{\bullet \ell } + \delta _\ell \textbf{1}_\ell - \varvec{\mu }_{\mathcal {T}_\ell })^{\text {T}} {{\varvec{V}}_\ell ^{(R)}}^{-1} (\widehat{\varvec{\beta }}^{(R)}_{\bullet \ell } + \delta _\ell \textbf{1}_\ell - \varvec{\mu }_{\mathcal {T}_\ell }),\ \text {subject to}\ \Vert \varvec{\mu } \Vert _0 \le s, \end{aligned}$$

where $n_0$ is the total sample size across studies, $\textbf{1}_\ell$ is a $K_\ell$-vector of ones, $\varvec{\mu }_{\mathcal {T}_\ell }$ is the subset of $\varvec{\mu }$ for features in $\mathcal {T}_\ell$, and $\Vert \varvec{\mu } \Vert _0=\sum \nolimits _{k=1}^{K_0} I(\mu _{k}\ne 0)$ is the $\ell _0$ norm of $\varvec{\mu }$. The form of $\mathcal {L}(\varvec{\mu })$ is motivated by the inverse-variance estimator commonly used in meta-analyses [66], and the cardinality constraint with the sparsity level *s* is formulated as in the classical best subset selection problem [34, 35]. By noting the relationship between AA and RA association coefficients $\beta ^{(R)}_{k\ell } = \beta _{k\ell } - \beta _{R\ell }$ ($k\in \mathcal {T}_\ell$), we introduce a hyperparameter $\delta _\ell$ in each study $\ell$ to offset $\beta _{R\ell }$ in $\beta ^{(R)}_{k\ell }$. By varying the value of $\delta _\ell$, one essentially traverses possible versions of putative AA association coefficients in study $\ell$. Given $\delta _1, \ldots , \delta _L$ and *s*, we solve the best subset selection problem (4) using the polynomial algorithm [67, 68]. The optimal values of the hyperparameters are determined by minimizing BIC defined as

5 $$\begin{aligned} \text {BIC}(s, \delta _1, \ldots , \delta _L) = n_0^{-1} \sum \limits _{\ell =1}^L (\widehat{\varvec{\beta }}^{(R)}_{\bullet \ell } + {\delta }_{\ell }\textbf{1}_{\ell } - \widetilde{\varvec{\mu }}_{\mathcal {T}_\ell } )^{\text {T}} {{\varvec{V}}_\ell ^{(R)}}^{-1} (\widehat{\varvec{\beta }}^{(R)}_{\bullet \ell } + {\delta }_{\ell } \textbf{1}_{\ell } - \widetilde{\varvec{\mu }}_{\mathcal {T}_\ell } ) + \Vert \widetilde{\varvec{\mu }} \Vert _0 \frac{ \log n_0}{n_0}, \end{aligned}$$

where $\widetilde{\varvec{\mu }}$ is the estimate of $\varvec{\mu }$ under a specification of hyperparameters. Details on solving the best subset selection problem and hyperparameter tuning algorithms that utilized Powell’s algorithm [69], the successive parabolic interpolation (SPI) algorithm [70], and the golden section search algorithm [71], are provided in the Additional file 1: Supplementary Note A.

The $\widetilde{\varvec{\mu }}$ under the optimal hyperparameter values is the estimate of the most parsimonious version of the putative AA associations consistent across studies. The features with non-zero values in $\widetilde{\varvec{\mu }}$ are the driver signatures detected by Melody.

### Simulation strategy

We simulated microbiome data by mimicking the species composition patterns of the five metagenomics studies for CRC [3]. For each study, we first fit the generalized Dirichlet multinomial distribution [72] to the 401 species with a prevalence of at least 20% in the real data and obtained the maximum likelihood parameter estimates. Using the five sets of estimated parameters, we randomly generated independent samples of proportions for five studies from the generalized Dirichlet distribution. These samples served as the true proportions of the microbial features. The synthetic data closely resemble the structure of the real microbiome data (Additional file 1: Fig. S2). We considered two settings for the sample sizes of the five studies: $n=100, 120, 140, 160, 180$ for relatively large studies, and $n=20, 30, 40, 50, 60$ for small studies. We randomly assigned the samples into 50% cases and 50% controls. We varied the number of signatures from 4 (1% of the features) to 160 (40%). Half of the signatures were randomly selected from the set of relatively abundant features with an average proportion of at least $10^{-3}$ in the original real data, while the other half were randomly selected from the remaining features. We let *pos*% of the signatures have positive effects and $1-pos$% have negative effects. We considered three settings of *pos*%: $60\%$, $80\%$, and $100\%$, which reflect various degrees of sign imbalance among signatures. For each positive-effect signature, we increased their proportions in cases; for each negative-effect signature, we increased their proportions in controls. The increased fold for each signature was randomly sampled from Uniform(0, $\Delta$), where $\Delta =2$ under the large study setting and $\Delta =5$ under the small study setting. The larger effect size in the small study was chosen to make performance differences among the compared methods more discernible. Next, we normalized the proportions of all features in each sample to ensure that the total sum of all proportions is one. Notably, the proportions of all features are shifted after this step, but the features initially changed are considered the driver signatures with different underlying absolute abundances between the case and control groups. We then used the normalized proportions as multinomial parameters to sample count data. The sequencing depth for samples in each study was randomly drawn from the pool of sequencing depth in the corresponding CRC study. Within each study, we increased the sequencing depth of all samples in either case or control group by a factor *u* to reflect the potential uneven sequencing depth between the two groups of interest. We considered three settings of *u*: 0, 0.5, and 1. We simulated 100 datasets for each scenario and reported the average AUPRC for signature selection in the simulated data for each meta-analysis approach.

We conducted another set of simulation experiments for meta-analyzing studies with correlated samples. We first simulated independent samples as described above. We randomly picked two samples $X_{i1}$ and $X_{i2}$ to form cluster *i*. To introduce correlation between samples within a cluster, we sample baseline composition $X^{*}_{i}$ from the generalized Dirichlet distribution and merge this baseline composition into $X_{i1}$ and $X_{i2}$ and form the correlated samples $X^{*}_{i1} = (X^{*}_{i}+X_{i1})/2$ and $X^{*}_{i2}=(X^{*}_{i}+X_{i2})/2$ for cluster *i*. The case and control labels are randomly assigned to clusters. Differential abundance between cases and controls is introduced in the same manner as for independent samples.

### Datasets of the five metagenomics studies for CRC

All raw sequencing data across studies were reprocessed using the same bioinformatics pipeline for taxonomic profiling [3]. The species-level microbiome count data and the variable for CRC status were obtained from https://github.com/zellerlab/crc_meta. The five studies involve subjects from five different countries. The sample sizes of the studies are 109, 127, 120, 114, and 104 (Additional file 1: Table S1). We removed two samples with an outlier sequencing depth of less than 2000. Our main analysis was on $K=401$ species after removing species with a prevalence less than $20\%$ (prevalence was defined as the percentage of non-zero counts in all samples across studies). Additionally, we analyzed the data with all $K=849$ species and with $K=267$ species under order *Clostridiales* to assess the stability of signature selection.

### Datasets of the eight microbiome-metabolome studies

In all studies, the microbiome and metabolome were profiled from the same fecal samples. The datasets undergo curation and processing as part of an established effort [73]. The genus-level microbiome count data and the variables for metabolites and disease status were obtained from https://github.com/borenstein-lab/microbiome-metabolome-curated-data. We used the eight adult cohorts from this resource with sample sizes of 96, 294, 240, 220, 444, 382, 287, and 164. There is notable heterogeneity among studies in study designs, microbiome sequencing methods, and metabolite measurements (Additional file 1: Table S3). Our analysis focused on 450 metabolites with study-specific prevalence $\ge 10\%$ in at least two studies and 101 genera with study-specific prevalence $\ge 10\%$ in at least one study. We applied rank-based inverse normal transformation to the metabolite variable. We performed Melody and MMUPHin to discover genus signatures for each metabolite. In both methods, we analyze each metabolite variable as the covariate of interest while adjusting the disease status as a confounder when generating summary statistics within individual studies.

### R packages utilized

*MMUPHin* (v1.14.0), *ALDEx2* (v1.32.0), *ANCOMBC* (v2.2.2), *coin* (v1.4-2), *glmnet* (v4.1-8), *brglm2* (v 0.9.2), *abess* (v0.4.8), *mlr3* (v0.16.1).

## Supplementary Information

Additional file 1: Supplementary Notes A and B, Figs S1-S14, and Tables S1, S3.

Additional file 2: Tables S2, S4, S5.

## Data Availability

This study made use of 13 publicly available datasets. The five metagenomics data for colorectal cancer were obtained from https://github.com/zellerlab/crc_meta [74] and the eight microbiome-metabolome data were obtained from https://github.com/borenstein-lab/microbiome-metabolome-curated-data [75]. All raw data and processed data used for analysis are also available at https://github.com/ZjpWei/Melody [76]. Melody is implemented in the R package miMeta publicly available at https://github.com/ZjpWei/miMeta [77]. The site also contains a complete set of manuals and instructions. The scripts generating reported results are deposited at Github https://github.com/ZjpWei/Melody [76] and Zenodo https://doi.org/10.5281/zenodo.15666043 [78] under the GPL-3.0 license.

## References

[1] Tringe SG, Rubin EM. Metagenomics: DNA sequencing of environmental samples. Nat Rev Genet. 2005;6(11):805–14. 16304596 10.1038/nrg1709

[2] Duvallet C, Gibbons SM, Gurry T, Irizarry RA, Alm EJ. Meta-analysis of gut microbiome studies identifies disease-specific and shared responses. Nat Commun. 2017;8(1):1784. 29209090 10.1038/s41467-017-01973-8 PMC5716994

[3] Wirbel J, Pyl PT, Kartal E, Zych K, Kashani A, Milanese A, et al. Meta-analysis of fecal metagenomes reveals global microbial signatures that are specific for colorectal cancer. Nat Med. 2019;25:679–89. 30936547 10.1038/s41591-019-0406-6 PMC7984229

[4] Muller E, Algavi YM, Borenstein E. A meta-analysis study of the robustness and universality of gut microbiome-metabolome associations. Microbiome. 2021;9(1):1–18. 34641974 10.1186/s40168-021-01149-z PMC8507343

[5] Ma S, Shungin D, Mallick H, Schirmer M, Nguyen LH, Kolde R, et al. Population structure discovery in meta-analyzed microbial communities and inflammatory bowel disease using MMUPHin. Genome Biol. 2022;23(1):1–31. 36192803 10.1186/s13059-022-02753-4 PMC9531436

[6] Hamady M, Knight R. Microbial community profiling for human microbiome projects: tools, techniques, and challenges. Genome Res. 2009;19(7):1141–52. 19383763 10.1101/gr.085464.108 PMC3776646

[7] Gloor GB, Wu JR, Pawlowsky-Glahn V, Egozcue JJ. It’s all relative: analyzing microbiome data as compositions. Ann Epidemiol. 2016;26(5):322–9. 27143475 10.1016/j.annepidem.2016.03.003

[8] Gloor GB, Macklaim JM, Pawlowsky-Glahn V, Egozcue JJ. Microbiome datasets are compositional: and this is not optional. Front Microbiol. 2017;8:2224. 29187837 10.3389/fmicb.2017.02224 PMC5695134

[9] Fredricks DN, Fiedler TL, Thomas KK, Oakley BB, Marrazzo JM. Targeted PCR for detection of vaginal bacteria associated with bacterial vaginosis. J Clin Microbiol. 2007;45(10):3270–6. 17687006 10.1128/JCM.01272-07 PMC2045326

[10] Ryu H, Henson M, Elk M, Toledo-Hernandez C, Griffith J, Blackwood D, et al. Development of quantitative PCR assays targeting the 16S rRNA genes of Enterococcus spp. and their application to the identification of Enterococcus species in environmental samples. Appl Environ Microbiol. 2013;79(1):196–204. 23087032 10.1128/AEM.02802-12 PMC3536114

[11] Vandeputte D, Kathagen G, D’hoe K, Vieira-Silva S, Valles-Colomer M, Sabino J, et al. Quantitative microbiome profiling links gut community variation to microbial load. Nature. 2017;551(7681):507–11. 29143816 10.1038/nature24460

[12] McLaren MR, Willis AD, Callahan BJ. Consistent and correctable bias in metagenomic sequencing experiments. Elife. 2019;8:e46923. 31502536 10.7554/eLife.46923 PMC6739870

[13] Morton JT, Marotz C, Washburne A, Silverman J, Zaramela LS, Edlund A, et al. Establishing microbial composition measurement standards with reference frames. Nat Commun. 2019;10(1):2719. 31222023 10.1038/s41467-019-10656-5 PMC6586903

[14] Hu Y, Satten GA, Hu YJ. LOCOM: A logistic regression model for testing differential abundance in compositional microbiome data with false discovery rate control. Proc Natl Acad Sci. 2022;119(30):e2122788119. 35867822 10.1073/pnas.2122788119 PMC9335309

[15] Zhou H, He K, Chen J, Zhang X. LinDA: linear models for differential abundance analysis of microbiome compositional data. Genome Biol. 2022;23(1):1–23. 35421994 10.1186/s13059-022-02655-5 PMC9012043

[16] Nixon MP, McGovern KC, Letourneau J, David LA, Lazar NA, Mukherjee S, Silverman JD. Scale reliant inference. 2022. arXiv preprint arXiv:2201.03616.

[17] Lin H, Peddada SD. Multigroup analysis of compositions of microbiomes with covariate adjustments and repeated measures. Nat Methods. 2024;21(1):83–91. 38158428 10.1038/s41592-023-02092-7 PMC10776411

[18] Clausen DS, Teichman S, Willis AD. Estimating fold changes from partially observed outcomes with applications in microbial metagenomics. 2024. arXiv preprint arXiv:2402.05231.

[19] Weiss S, Xu ZZ, Peddada S, Amir A, Bittinger K, Gonzalez A, et al. Normalization and microbial differential abundance strategies depend upon data characteristics. Microbiome. 2017;5:1–18. 28253908 10.1186/s40168-017-0237-y PMC5335496

[20] McMurdie PJ, Holmes S. Waste not, want not: why rarefying microbiome data is inadmissible. PLoS Comput Biol. 2014;10(4):e1003531. 24699258 10.1371/journal.pcbi.1003531 PMC3974642

[21] Willis AD. Rarefaction, alpha diversity, and statistics. Front Microbiol. 2019;10:2407. 31708888 10.3389/fmicb.2019.02407 PMC6819366

[22] Costea PI, Zeller G, Sunagawa S, Bork P. A fair comparison. Nat Methods. 2014;11(4):359. 24681719 10.1038/nmeth.2897

[23] Nearing JT, Douglas GM, Hayes MG, MacDonald J, Desai DK, Allward N, et al. Microbiome differential abundance methods produce different results across 38 datasets. Nat Commun. 2022;13(1):342. 35039521 10.1038/s41467-022-28034-z PMC8763921

[24] Schloss PD. Removal of rare amplicon sequence variants from 16S rRNA gene sequence surveys biases the interpretation of community structure data. bioRxiv. 2020. 10.1101/2020.12.11.422279.

[25] Gibbons SM, Duvallet C, Alm EJ. Correcting for batch effects in case-control microbiome studies. PLoS Comput Biol. 2018;14(4):e1006102. 29684016 10.1371/journal.pcbi.1006102 PMC5940237

[26] Weiss S, Amir A, Hyde ER, Metcalf JL, Song SJ, Knight R. Tracking down the sources of experimental contamination in microbiome studies. Genome Biol. 2014;15(12):1–3. 10.1186/s13059-014-0564-2 PMC4311479 25608874

[27] Nguyen NH, Smith D, Peay K, Kennedy P. Parsing ecological signal from noise in next generation amplicon sequencing. New Phytol. 2015;205(4):1389–93. 24985885 10.1111/nph.12923

[28] Leek JT, Scharpf RB, Bravo HC, Simcha D, Langmead B, Johnson WE, et al. Tackling the widespread and critical impact of batch effects in high-throughput data. Nat Rev Genet. 2010;11(10):733–9. 20838408 10.1038/nrg2825 PMC3880143

[29] Nygaard V, Rødland EA, Hovig E. Methods that remove batch effects while retaining group differences may lead to exaggerated confidence in downstream analyses. Biostatistics. 2016;17(1):29–39. 26272994 10.1093/biostatistics/kxv027 PMC4679072

[30] Goh WWB, Wang W, Wong L. Why batch effects matter in omics data, and how to avoid them. Trends Biotechnol. 2017;35(6):498–507. 28351613 10.1016/j.tibtech.2017.02.012

[31] Evangelou E, Ioannidis JP. Meta-analysis methods for genome-wide association studies and beyond. Nat Rev Genet. 2013;14(6):379–89. 23657481 10.1038/nrg3472

[32] Lin DY, Tang ZZ. A general framework for detecting disease associations with rare variants in sequencing studies. Am J Hum Genet. 2011;89(3):354–67. 21885029 10.1016/j.ajhg.2011.07.015 PMC3169821

[33] Tang ZZ, Lin DY. Meta-analysis of sequencing studies with heterogeneous genetic associations. Genet Epidemiol. 2014;38(5):389–401. 24799183 10.1002/gepi.21798 PMC4157393

[34] Beale EML, Kendall MG, Mann D. The discarding of variables in multivariate analysis. Biometrika. 1967;54(3–4):357–66. 6063999

[35] Hocking RR, Leslie R. Selection of the best subset in regression analysis. Technometrics. 1967;9(4):531–40.

[36] Mallick H, Rahnavard A, McIver LJ, Ma S, Zhang Y, Nguyen LH, et al. Multivariable association discovery in population-scale meta-omics studies. PLoS Comput Biol. 2021;17(11):e1009442. 34784344 10.1371/journal.pcbi.1009442 PMC8714082

[37] Viechtbauer W. Conducting meta-analyses in R with the metafor package. J Stat Softw. 2010;36(3):1–48.

[38] Fernandes AD, Reid JN, Macklaim JM, McMurrough TA, Edgell DR, Gloor GB. Unifying the analysis of high-throughput sequencing datasets: characterizing RNA-seq, 16S rRNA gene sequencing and selective growth experiments by compositional data analysis. Microbiome. 2014;2:1–13. 24910773 10.1186/2049-2618-2-15 PMC4030730

[39] Susin A, Wang Y, Lê Cao KA, Calle ML. Variable selection in microbiome compositional data analysis. NAR Genomics Bioinforma. 2020;2(2):lqaa029. 10.1093/nargab/lqaa029 PMC7671404 33575585

[40] Tibshirani R. Regression shrinkage and selection via the lasso. J R Stat Soc Ser B Stat Methodol. 1996;58(1):267–88.

[41] Yu J, Feng Q, Wong SH, Zhang D, Yi Liang Q, Qin Y, et al. Metagenomic analysis of faecal microbiome as a tool towards targeted non-invasive biomarkers for colorectal cancer. Gut. 2017;66(1):70–8. 26408641 10.1136/gutjnl-2015-309800

[42] Vogtmann E, Hua X, Zeller G, Sunagawa S, Voigt AY, Hercog R, et al. Colorectal cancer and the human gut microbiome: reproducibility with whole-genome shotgun sequencing. PloS One. 2016;11(5):e0155362. 27171425 10.1371/journal.pone.0155362 PMC4865240

[43] Feng Q, Liang S, Jia H, Stadlmayr A, Tang L, Lan Z, et al. Gut microbiome development along the colorectal adenoma-carcinoma sequence. Nat Commun. 2015;6(1):6528. 25758642 10.1038/ncomms7528

[44] Zeller G, Tap J, Voigt AY, Sunagawa S, Kultima JR, Costea PI, et al. Potential of fecal microbiota for early-stage detection of colorectal cancer. Mol Syst Biol. 2014;10(11):766. 25432777 10.15252/msb.20145645 PMC4299606

[45] Benjamini Y, Hochberg Y. Controlling the false discovery rate: a practical and powerful approach to multiple testing. J R Stat Soc Ser B (Methodol). 1995;57(1):289–300.

[46] Zhao L, Zhang X, Zhou Y, Fu K, Lau HCH, Chun TWY, et al. Parvimonas micra promotes colorectal tumorigenesis and is associated with prognosis of colorectal cancer patients. Oncogene. 2022;41(36):4200–10. 35882981 10.1038/s41388-022-02395-7 PMC9439953

[47] Wong SH, Yu J. Gut microbiota in colorectal cancer: mechanisms of action and clinical applications. Nat Rev Gastroenterol Hepatol. 2019;16(11):690–704. 31554963 10.1038/s41575-019-0209-8

[48] Erawijantari PP, Mizutani S, Shiroma H, Shiba S, Nakajima T, Sakamoto T, et al. Influence of gastrectomy for gastric cancer treatment on faecal microbiome and metabolome profiles. Gut. 2020;69(8):1404–15. 31953253 10.1136/gutjnl-2019-319188 PMC7398469

[49] Yachida S, Mizutani S, Shiroma H, Shiba S, Nakajima T, Sakamoto T, et al. Metagenomic and metabolomic analyses reveal distinct stage-specific phenotypes of the gut microbiota in colorectal cancer. Nat Med. 2019;25(6):968–76. 31171880 10.1038/s41591-019-0458-7

[50] Kim M, Vogtmann E, Ahlquist DA, Devens ME, Kisiel JB, Taylor WR, et al. Fecal metabolomic signatures in colorectal adenoma patients are associated with gut microbiota and early events of colorectal cancer pathogenesis. MBio. 2020;11(1):10–1128. 10.1128/mBio.03186-19 PMC7029137 32071266

[51] Franzosa EA, Sirota-Madi A, Avila-Pacheco J, Fornelos N, Haiser HJ, Reinker S, et al. Gut microbiome structure and metabolic activity in inflammatory bowel disease. Nat Microbiol. 2019;4(2):293–305. 30531976 10.1038/s41564-018-0306-4 PMC6342642

[52] Mars RA, Yang Y, Ward T, Houtti M, Priya S, Lekatz HR, et al. Longitudinal multi-omics reveals subset-specific mechanisms underlying irritable bowel syndrome. Cell. 2020;182(6):1460–73. 32916129 10.1016/j.cell.2020.08.007 PMC8109273

[53] Lloyd-Price J, Arze C, Ananthakrishnan AN, Schirmer M, Avila-Pacheco J, Poon TW, et al. Multi-omics of the gut microbial ecosystem in inflammatory bowel diseases. Nature. 2019;569(7758):655–62. 31142855 10.1038/s41586-019-1237-9 PMC6650278

[54] Wang X, Yang S, Li S, Zhao L, Hao Y, Qin J, et al. Aberrant gut microbiota alters host metabolome and impacts renal failure in humans and rodents. Gut. 2020;69(12):2131–42. 32241904 10.1136/gutjnl-2019-319766 PMC7677483

[55] Poyet M, Groussin M, Gibbons SM, Avila-Pacheco J, Jiang X, Kearney SM, et al. A library of human gut bacterial isolates paired with longitudinal multiomics data enables mechanistic microbiome research. Nat Med. 2019;25(9):1442–52. 31477907 10.1038/s41591-019-0559-3

[56] Wishart DS, Feunang YD, Marcu A, Guo AC, Liang K, Vázquez-Fresno R, et al. HMDB 4.0: the human metabolome database for 2018. Nucleic Acids Res. 2018;46(D1):D608–17. 29140435 10.1093/nar/gkx1089 PMC5753273

[57] Duncan SH, Louis P, Flint HJ. Lactate-utilizing bacteria, isolated from human feces, that produce butyrate as a major fermentation product. Appl Environ Microbiol. 2004;70(10):5810–7. 15466518 10.1128/AEM.70.10.5810-5817.2004 PMC522113

[58] Louis P, Hold GL, Flint HJ. The gut microbiota, bacterial metabolites and colorectal cancer. Nat Rev Microbiol. 2014;12(10):661–72. 25198138 10.1038/nrmicro3344

[59] Machiels K, Joossens M, Sabino J, De Preter V, Arijs I, Eeckhaut V, et al. A decrease of the butyrate-producing species Roseburia hominis and Faecalibacterium prausnitzii defines dysbiosis in patients with ulcerative colitis. Gut. 2014;63(8):1275–83. 24021287 10.1136/gutjnl-2013-304833

[60] Org E, Blum Y, Kasela S, Mehrabian M, Kuusisto J, Kangas AJ, et al. Relationships between gut microbiota, plasma metabolites, and metabolic syndrome traits in the METSIM cohort. Genome Biol. 2017;18:1–14. 28407784 10.1186/s13059-017-1194-2 PMC5390365

[61] Zeng Q, Li D, He Y, Li Y, Yang Z, Zhao X, et al. Discrepant gut microbiota markers for the classification of obesity-related metabolic abnormalities. Sci Rep. 2019;9(1):13424. 31530820 10.1038/s41598-019-49462-w PMC6748942

[62] Koeth RA, Levison BS, Culley MK, Buffa JA, Wang Z, Gregory JC, et al. -Butyrobetaine is a proatherogenic intermediate in gut microbial metabolism of L-carnitine to TMAO. Cell Metab. 2014;20(5):799–812. 10.1016/j.cmet.2014.10.006 PMC4255476 25440057

[63] Min Y, Ma X, Sankaran K, Ru Y, Chen L, Baiocchi M, et al. Sex-specific association between gut microbiome and fat distribution. Nat Commun. 2019;10(1):2408. 31160598 10.1038/s41467-019-10440-5 PMC6546740

[64] Borenstein M, Hedges LV, Higgins JP, Rothstein HR. Introduction to meta-analysis. Wiley; 2021.

[65] Kosmidis I, Kenne Pagui EC, Sartori N. Mean and median bias reduction in generalized linear models. Stat Comput. 2020;30(1):43–59.

[66] He Q, Zhang HH, Avery CL, Lin D. Sparse meta-analysis with high-dimensional data. Biostatistics. 2016;17(2):205–20. 26395907 10.1093/biostatistics/kxv038 PMC4834947

[67] Zhu J, Wen C, Zhu J, Zhang H, Wang X. A polynomial algorithm for best-subset selection problem. Proc Natl Acad Sci. 2020;117(52):33117–23. 33328272 10.1073/pnas.2014241117 PMC7777147

[68] Zhu J, Wang X, Hu L, Huang J, Jiang K, Zhang Y, et al. abess: a fast best-subset selection library in python and R. J Mach Learn Res. 2022;23(1):9206–12.

[69] Powell MJ. An efficient method for finding the minimum of a function of several variables without calculating derivatives. Comput J. 1964;7(2):155–62.

[70] Heath MT. Scientific computing: an introductory survey, revised second edition. Philadelphia, PA: SIAM; 2018.

[71] Kiefer J. Sequential minimax search for a maximum. Proc Am Math Soc. 1953;4(3):502–6.

[72] Tang ZZ, Chen G. Zero-inflated generalized Dirichlet multinomial regression model for microbiome compositional data analysis. Biostatistics. 2019;20(4):698–713. 29939212 10.1093/biostatistics/kxy025 PMC7410344

[73] Muller E, Algavi YM, Borenstein E. The gut microbiome-metabolome dataset collection: a curated resource for integrative meta-analysis. npj Biofilms Microbiomes. 2022;8(1):79. 36243731 10.1038/s41522-022-00345-5 PMC9569371

[74] Wirbel J. Colorectal cancer meta-analysis. Github. 2019. https://github.com/zellerlab/crc_meta. Accessed 01 Dec 2023.

[75] Borenstein E. The curated gut microbiome metabolome data resource. Github. 2024. https://github.com/borensteinlab/microbiome-metabolome-curated-data. Accessed 01 Jan 2024.

[76] Wei Z, Chen G, Tang ZZ. Melody: meta-analysis of microbiome association studies for discovering generalizable microbial signatures. Github. 2025. https://github.com/ZjpWei/Melody. Accessed 01 Jun 2025. 10.1186/s13059-025-03721-4 PMC12359909 40826361

[77] Wei Z, Chen G, Tang ZZ. miMeta R package. Github. 2025. https://github.com/ZjpWei/miMeta. Accessed 01 May 2025.

[78] Wei Z, Chen G, Tang ZZ. Melody: meta-analysis of microbiome association studies for discovering generalizable microbial signatures. Zenodo. 2025. 10.5281/zenodo.15666043. 10.1186/s13059-025-03721-4 PMC12359909 40826361

