# Supplementary material for: Melody: meta-analysis of microbiome association studies for discovering generalizable microbial signatures
Source: Genome Biol. 2025 Aug 18;26:245. doi: 10.1186/s13059-025-03721-4 (PMC12359909; doi:10.1186/s13059-025-03721-4)
Supplement: Supplementary file 1 — Additional file 1: Supplementary Notes A and B, Figs S1-S14, and Tables S1, S3. [file 13059_2025_3721_MOESM1_ESM.pdf]

Supplementary Information For:

Melody: meta-analysis of microbiome association studies for discovering generalizable microbial signatures

Zhoujingpeng Wei, Guanhua Chen, and Zheng-Zheng Tang

Supplementary Note A: Additional Details of Melody

1. Generating summary statistics for studies with correlated samples

Suppose we have n clusters in the data. We let i index clusters ($i = 1, \ldots, n$) and t index correlated samples within a particular cluster ($t = 1 \ldots, T_i$). The notation for data is defined similarly as in Methods but replacing subscript i with it to index sample t within cluster i. The quasi-score function can be written as

$$\boldsymbol{S}(\boldsymbol{\theta}^{(R)}) = \sum_{i=1}^{n} \sum_{t=1}^{T_i} (\boldsymbol{Y}_{it} - N_{it}\boldsymbol{p}_{it}) \otimes \mathbf{X}_{it},$$

where $\boldsymbol{Y}_{it} = \{Y_{itk}\}_{k \in \backslash R}$, $\boldsymbol{p}_{it} = \{p_{itk}\}_{k \in \backslash R}$ and

$$p_{itk} = \frac{\exp(\mathbf{X}_{it}^{\mathrm{T}} \boldsymbol{\theta}_k^{(R)})}{1 + \sum_{j \in \backslash R} \exp(\mathbf{X}_{it}^{\mathrm{T}} \boldsymbol{\theta}_j^{(R)})}.$$

Let $\boldsymbol{S}_{\boldsymbol{\beta}}$ and $\boldsymbol{S}_{\boldsymbol{\gamma}}$ denote the components in $\boldsymbol{S}$ corresponding to $\boldsymbol{\beta}^{(R)}$ (RA association coefficients of interest) and $\boldsymbol{\gamma}^{(R)}$ (nuisance coefficients), respectively. We estimate $\widetilde{\boldsymbol{\theta}}^{(R)} = (\mathbf{0}, \widetilde{\boldsymbol{\gamma}}^{(R)})$ and construct $\widehat{\boldsymbol{\beta}}^{(R)}$, $\boldsymbol{\mathcal{I}}_{\boldsymbol{\beta}}$, and $\begin{bmatrix} \boldsymbol{\mathcal{I}}_{\boldsymbol{\beta\beta}} & \boldsymbol{\mathcal{I}}_{\boldsymbol{\beta\gamma}} \\ \boldsymbol{\mathcal{I}}_{\boldsymbol{\gamma\beta}} & \boldsymbol{\mathcal{I}}_{\boldsymbol{\gamma\gamma}} \end{bmatrix}$ using the same approaches as described in Methods. The sandwich estimator of the covariance of $\widehat{\boldsymbol{\beta}}^{(R)}$ can be modified to account for correlated samples as

$$\boldsymbol{V}^{(R)} = \boldsymbol{\mathcal{I}}_{\boldsymbol{\beta}}^{-1}(\widetilde{\boldsymbol{\theta}}^{(R)}) \left(\sum_{i=1}^{n} \left(\sum_{t=1}^{T_i} \boldsymbol{U}_{it}(\widetilde{\boldsymbol{\theta}}^{(R)}) \right) \left(\sum_{t=1}^{T_i} \boldsymbol{U}_{it}(\widetilde{\boldsymbol{\theta}}^{(R)}) \right)^{\mathrm{T}} \right) \boldsymbol{\mathcal{I}}_{\boldsymbol{\beta}}^{-1}(\widetilde{\boldsymbol{\theta}}^{(R)}),$$

where $\boldsymbol{U}_{it}(\widetilde{\boldsymbol{\theta}}^{(R)}) = \boldsymbol{S}_{\boldsymbol{\beta},it}(\widetilde{\boldsymbol{\theta}}^{(R)}) - \boldsymbol{\mathcal{I}}_{\boldsymbol{\beta\gamma}}(\widetilde{\boldsymbol{\theta}}^{(R)}) \boldsymbol{\mathcal{I}}_{\boldsymbol{\gamma\gamma}}^{-1}(\widetilde{\boldsymbol{\theta}}^{(R)}) \boldsymbol{S}_{\boldsymbol{\gamma},it}(\widetilde{\boldsymbol{\theta}}^{(R)})$, $\boldsymbol{S}_{\boldsymbol{\beta},it}$ and $\boldsymbol{S}_{\boldsymbol{\gamma},it}$ are the itth summand of $\boldsymbol{S}_{\boldsymbol{\beta}}$ and $\boldsymbol{S}_{\boldsymbol{\gamma}}$ respectively.

2. Solving the best subset selection problem

Given the specified hyperparameters, the optimization problem (4) in Methods can be conveniently solved by a best-subset selection solver after reformatting the $\mathcal{L}(\boldsymbol{\mu})$ objective function as $\|\breve{\boldsymbol{Y}} - \breve{\mathbf{X}}\boldsymbol{\mu}\|_2^2$ with the following definition

$$\breve{\boldsymbol{Y}} = \begin{pmatrix} \boldsymbol{\Sigma}_1 \widehat{\boldsymbol{\beta}}_{\bullet 1}^{(R)} + \delta_1 \boldsymbol{\Sigma}_1 \mathbf{1}_1 \\ \vdots \\ \boldsymbol{\Sigma}_L \widehat{\boldsymbol{\beta}}_{\bullet L}^{(R)} + \delta_L \boldsymbol{\Sigma}_L \mathbf{1}_L \end{pmatrix}, \quad \breve{\mathbf{X}} = \begin{pmatrix} \boldsymbol{\Sigma}_1 \mathbb{I}_1 \\ \vdots \\ \boldsymbol{\Sigma}_L \mathbb{I}_L \end{pmatrix}.$$

For $\ell = 1, \ldots, L$, the $\boldsymbol{\Sigma}_\ell$ is the square root of the inverse of $n_0 \boldsymbol{V}_\ell^{(R)}$ (i.e., $\boldsymbol{\Sigma}_\ell \boldsymbol{\Sigma}_\ell = n_0^{-1} \boldsymbol{V}_\ell^{(R)^{-1}}$) and $\mathbb{I}_\ell$ is a mapping matrix with dimension $K_\ell \times K_0$. The (j, k)th element of $\mathbb{I}_\ell$ equals 1 if the jth feature in $\widehat{\boldsymbol{\beta}}_{\bullet \ell}^{(R)}$ is mapped to the kth feature in $\boldsymbol{\mu}$, otherwise, the element equals 0. We employ the R package *abess*[1] to solve the best-subset selection problem with the outcome variable $\breve{\boldsymbol{Y}}$ and design matrix $\breve{\mathbf{X}}$.

3. Hyperparameter tuning algorithms

We search the optimal values for the hyperparameter $\boldsymbol{\delta} = (\delta_1, \ldots, \delta_L)$, and s by minimizing the BIC defined in formula (5) in Methods. We developed Algorithm 1 to search for the optimal $\boldsymbol{\delta}$ given a subset size s that minimizes the deviance in BIC denoted as $\text{Dev}(\boldsymbol{\delta} \mid s)$, and Algorithm 2 to jointly search for the optimal combination of $\boldsymbol{\delta}$ and s to minimize BIC. Algorithm 1 is based on Powell's algorithm[2] and the successive parabolic interpolation (SPI) algorithm[3]. Algorithm 2 is based on the golden section search algorithm[4].

Algorithm 1 Search for optimal values of $\boldsymbol{\delta}$ under a given subset size s

---

Require: tol, NMAX
- Initialize $\boldsymbol{\delta}^0 = \{\delta_\ell^0\}_{\ell=1}^L$, where δ_ℓ^0 is the median of all estimates in $\widehat{\beta}_{\bullet\ell}^{(R)}$
- Initialize a list of directions $\{\boldsymbol{v}_1, \ldots, \boldsymbol{v}_L\}$, where $\boldsymbol{v}_\ell$ is in the ℓ-th coordinate direction
- Initialize $N.iter = 0$
- **while** $N.iter <$ NMAX **do**
  - $N.iter = N.iter + 1$
  - **for** $\ell = 1, \ldots, L$ **do**
    - Use SPI to minimize $\text{Dev}(\boldsymbol{\delta}|s)$ along the line through $\boldsymbol{\delta}^{\ell-1}$ in the direction of $\boldsymbol{v}_\ell$
    - Set the minimum point to be $\boldsymbol{\delta}^\ell$
  - **end for**
  - Set $\boldsymbol{v}_{L+1} = \boldsymbol{\delta}^0 - \boldsymbol{\delta}^L$
  - Use SPI to minimize $\text{Dev}(\boldsymbol{\delta}|s)$ along the line through $\boldsymbol{\delta}^0$ in the direction of $\boldsymbol{v}_{L+1}$
  - Set the minimum point to be $\boldsymbol{\delta}^{L+1}$
  - **if** $|\boldsymbol{\delta}^{L+1} - \boldsymbol{\delta}^0| > tol$ **then**
    - Set $\boldsymbol{\delta}^0 = \boldsymbol{\delta}^{L+1}$
    - **if** $N.iter$ is not a multiplier of L **then**
      - Delete the direction that contribute the most to $\boldsymbol{v}^{L+1}$ from the direction list
      - Add $\boldsymbol{v}^{L+1}$ to the direction list
    - **else**
      - Reset the list of directions to the initial value
    - **end if**
  - **else**
    - Converge and stop
  - **end if**
- **end while**
- **return** optimal values $\boldsymbol{\delta}^{L+1}$, the minimized deviance and BIC value under the given s

---

Algorithm 2 Joint search for optimal values of $\boldsymbol{\delta}$ and s

Require: subset size search bound $s^{(\min)}$ and $s^{(\max)}$

Initialize $s^{(1)} = [0.618s^{(\min)} + 0.382s^{(\max)}]$ and $s^{(2)} = [0.382s^{(\min)} + 0.618s^{(\max)}]$

Run Algorithm 1 to obtain the minimized BIC under $s^{(1)}$ and $s^{(2)}$, respectively.

Set the BIC_1 and BIC_2 to the minimized BIC values under $s^{(1)}$ and $s^{(2)}$ respectively.

while $s^{(1)} \neq s^{(2)}$ **do**

  if $\text{BIC}_1 \leq \text{BIC}_2$ **then**

    $s^{(\max)} = s^{(2)}$, $s^{(2)} = s^{(1)}$, $s^{(1)} = [0.618s^{(\min)} + 0.382s^{(\max)}]$, $\text{BIC}_2 = \text{BIC}_1$

    Run Algorithm 1 to obtain the optimal $\boldsymbol{\delta}^*$ and the minimized BIC under $s^{(1)}$.

    Set BIC_1 to the minimized BIC value under $s^{(1)}$.

  else

    $s^{(\min)} = s^{(1)}$, $s^{(1)} = s^{(2)}$, $s^{(2)} = [0.382s^{(\min)} + 0.618s^{(\max)}]$, $\text{BIC}_1 = \text{BIC}_2$

    Run Algorithm 1 to obtain the optimal $\boldsymbol{\delta}^*$ and minimized BIC under $s^{(2)}$.

    Set BIC_2 to the minimized BIC value under $s^{(2)}$.

  end if

end while

return optimal values are $(\boldsymbol{\delta}^*, s^{(1)})$ and the minimized BIC is BIC_1

Note: the $[x]$ gives the nearest integer of x

Supplementary Note B: AA-based Simulation Strategy

We conducted additional simulation studies that utilize a different way to simulate microbiome data. To simulate microbiome data for study ℓ ($\ell = 1, \ldots, L$), we followed these steps:

1. For each sample i, simulate vector of proportions $P_i = \{P_{ik}\}_k$ from the generalized Dirichlet distribution with parameters estimated from the ℓth microbiome template dataset. This step is the same as in the other simulation strategy.

2. Simulate total microbial load T_i from negative binomial distribution using R function rnbinom with arguments mu=1e8 and size=0.5.

3. Construct AA: $A_{ik} = P_{ik}T_i$.

4. Randomly select driver signatures and add differential AA signal between two groups using spike-in as in the other simulation strategy, where Δ controls the strength of the association signal.

5. Sample multiplicative feature-specific measurement bias b_k from log-normal(0, σ_ℓ^2). We set $\sigma_\ell = 0.2 \times \ell^B$ to reflect different variability of feature-specific measurement bias across studies, where B controls the study-level batch effect.

6. Apply multiplicative bias: $A^*_{ik} = A_{ik}b_k$.

7. Generate observed counts from Multinomial(N_i, P^*_i), where $P^*_i = \{P^*_{ik}\}_k$ and $P^*_{ik} = A^*_{ik}/\Sigma_j A^*_{ij}$. The specification of N_i is the same as the other simulation strategy.

We considered various degrees of sign imbalance among signatures ($pos\% = 60\%, 80\%, 100\%$) and degrees of sequencing depth unevenness between the two groups of interest ($u = 0, 0.5, 1$). Under each of these scenarios, we changed four factors: (1) number of signatures, ranging from 4 (1% of the features) to 160 (40%), with the default value set to 40 (10%). (2) effect

size Δ, ranging from 1 to 3, with the default value set to 2. (3) number of simulated studies, ranging from 3 to 7, with the default value set to 5. The sample size of study ℓ is set to $n = 80 + 20 \times \ell$. (4) levels of study-level batch effect B, ranging from 0.5, 1, 1.5, 2, with the default value set to 1. While varying one of these four factors, we keep the other factors at the default values. We simulated 100 datasets for each setting and reported the average AUPRC for signature selection in the simulated data for each meta-analysis approach.

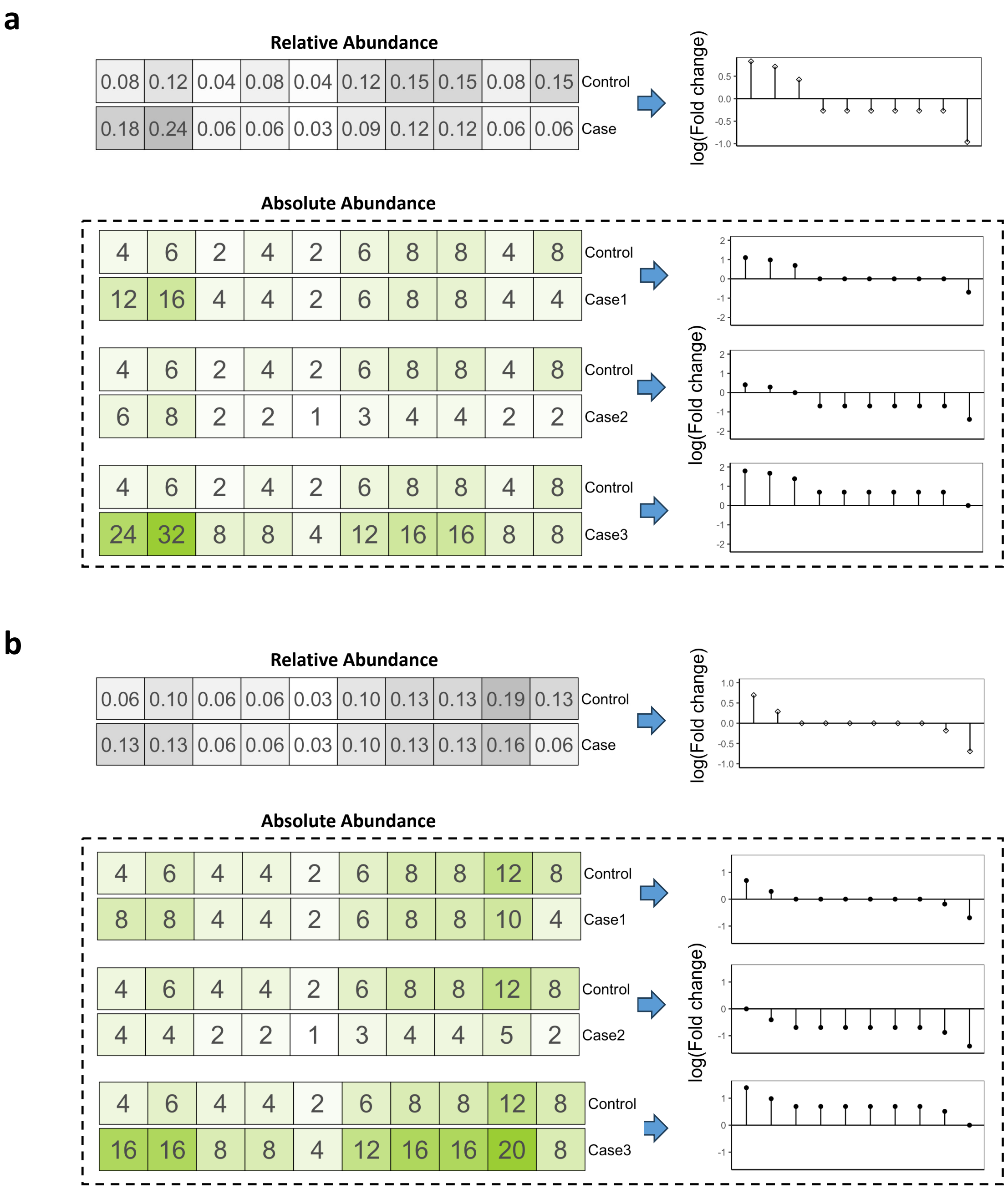

Fig. S1: Illustration of driver signatures in various scenarios of absolute abundance (AA) association profile compatible with the same relative abundance (RA) association profile. Section **a** illustrates a scenario where the RA association profile is not sparse, while section **b** depicts a sparse RA association profile. In each section, the upper panel displays the RA levels of 10 microbial features in case and control groups (left) and the corresponding RA association profile (right). The lower panel presents three different AA scenarios that yield the same RA profile (left) and their corresponding AA association profiles (right). For simplicity, the AA levels for the control group are held constant across the three scenarios. Driver signatures are the features with nonzero effects in the sparsest AA association profile (i.e., Case 1 vs. Control). In section **a**, driver signatures differ from the set of features with nonzero RA associations, whereas in section **b**, they coincide.

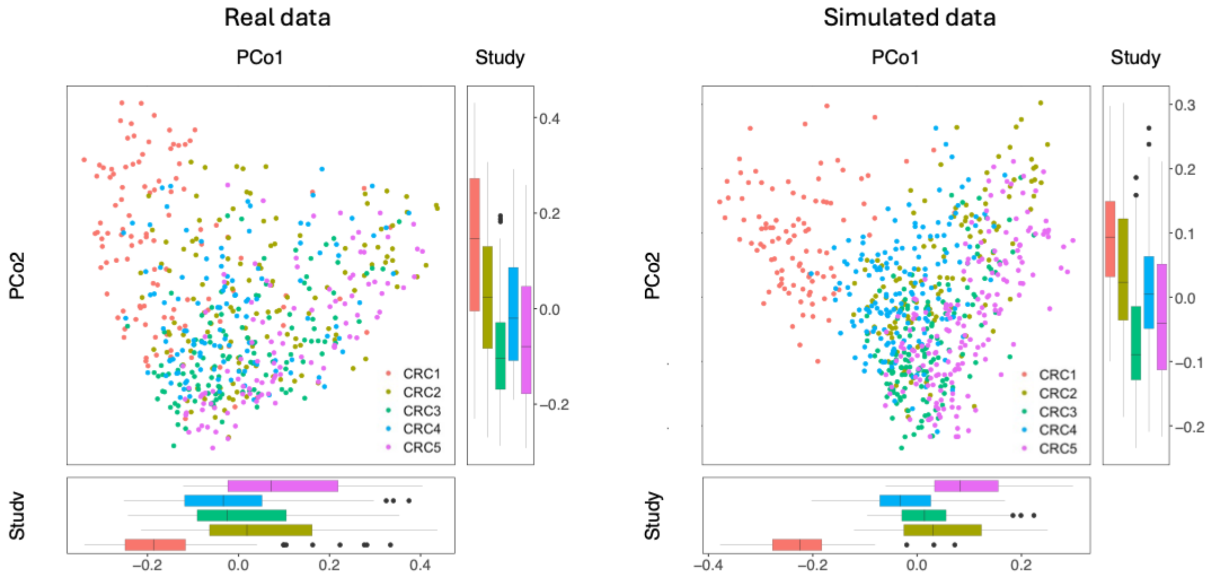

Fig. S2: Comparison of the structure of the real data and simulated data. Left: Principal coordinates analysis based on the species composition of all samples of the five metagenomics studies for colorectal cancer (CRC). Right: Principal coordinates analysis based on the simulated microbiome data of five studies that mimic the real data. Bray-Curtis dissimilarity is used in the principal coordinates analysis. Box plots represent differences among the five studies. Each box indicates the median (center line), the first and third quartiles (box edges), whiskers extending to 1.5 times the interquartile range, and individual outliers (dots). The box plots show strong batch effects across studies in both real and simulated data (Kruskal–Wallis rank-sum test $P < 10^{-20}$).

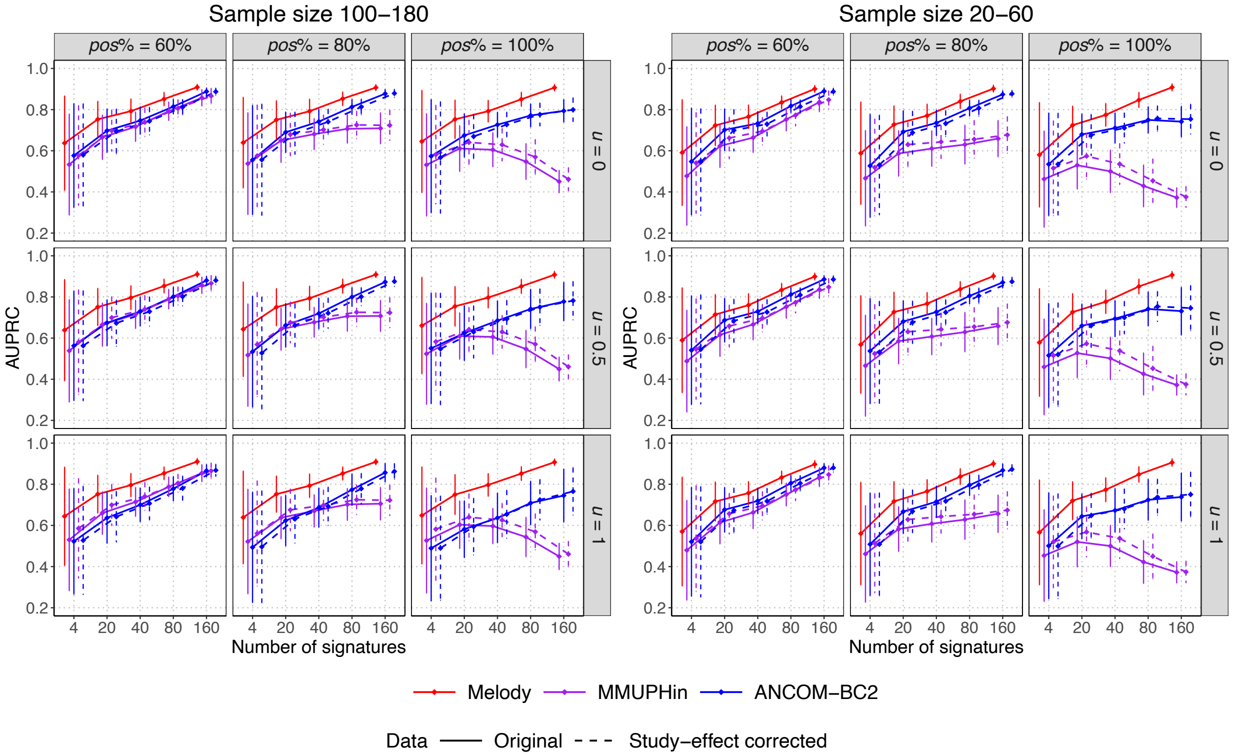

Fig. S3: Precision-recall evaluation of different meta-analysis signature selection approaches in simulated data with correlated samples from five studies. Melody was applied to the original data of the five studies, and the other methods were applied to both the original data and study-effect corrected data using the MMUPHin batch-effect correction algorithm. The x-axis represents the number of signatures. The left and right sections show the results under different sample size settings (the sample size range for the five studies is 100-180 or 20-60). Within each section, the columns of the plots represent various degrees of sign imbalance among signatures ($pos\%$ denotes the percentage of signatures with positive effects); the rows of the plots represent various degrees of sequencing depth unevenness between the two groups of interest (u denotes the sequencing depth relative change ratio for the group with higher sequencing depth, and 0 indicates no unevenness). Each panel displays the mean area under the precision-recall curve (AUPRC) with $\pm$ standard errors (indicated by error bars) based on 100 simulation replicates.

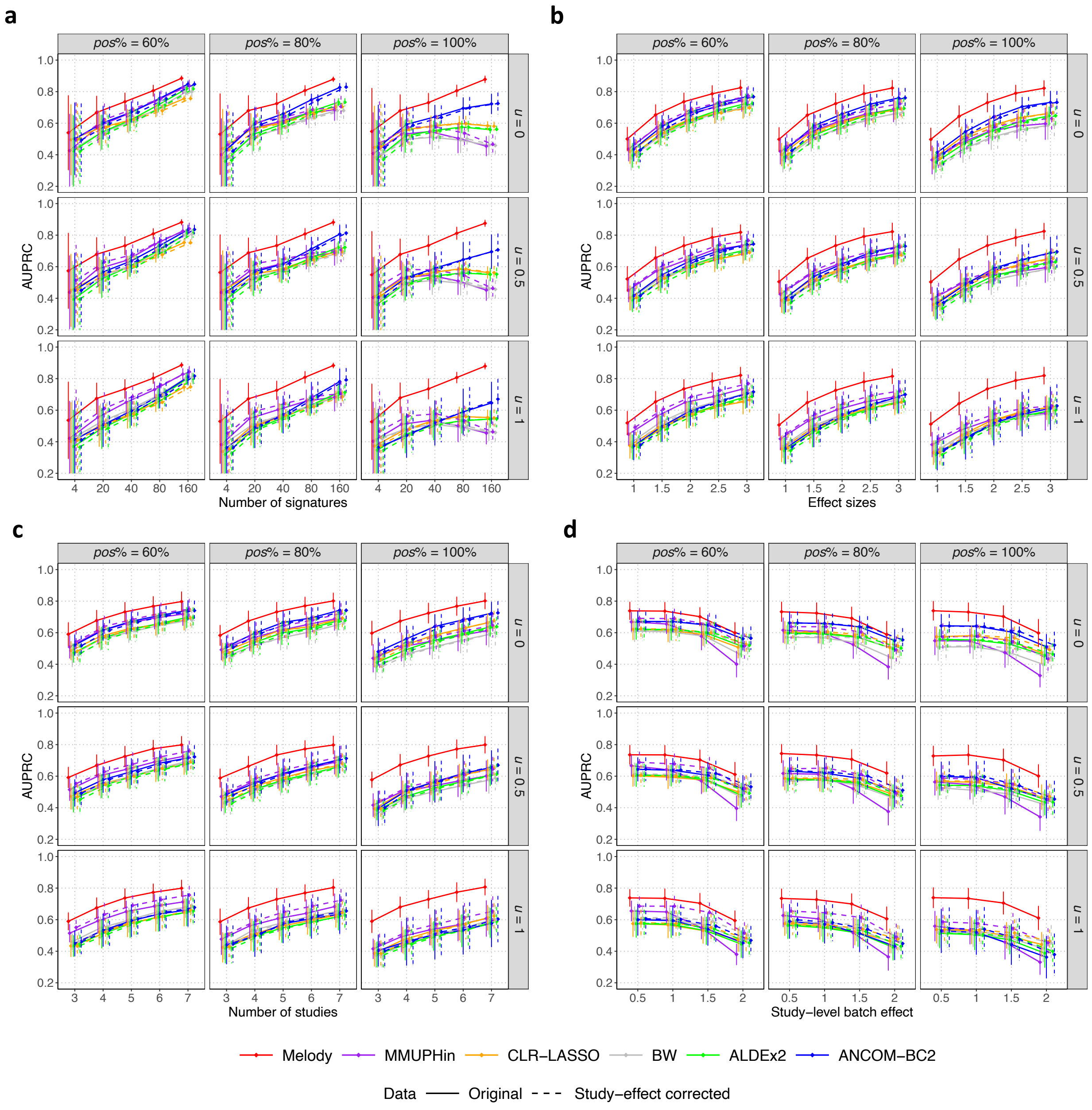

Fig. S4: Precision-recall evaluation of different meta-analysis signature selection approaches in the AA-based simulation strategy. Four different factors are varied and shown on the x-axis in sections **a-d**. Melody was applied to the original data of the five studies, and the other methods were applied to both the original data and study-effect corrected data using the MMUPHin batch-effect correction algorithm. Within each section, the columns of the plots represent various degrees of sign imbalance among signatures ($pos\%$ denotes the percentage of signatures with positive effects); the rows of the plots represent various degrees of sequencing depth unevenness between the two groups of interest (u denotes the sequencing depth relative change ratio for the group with higher sequencing depth, and 0 indicates no unevenness). Each panel displays the mean area under the precision-recall curve (AUPRC) with ± standard errors (indicated by error bars) based on 100 simulation replicates.

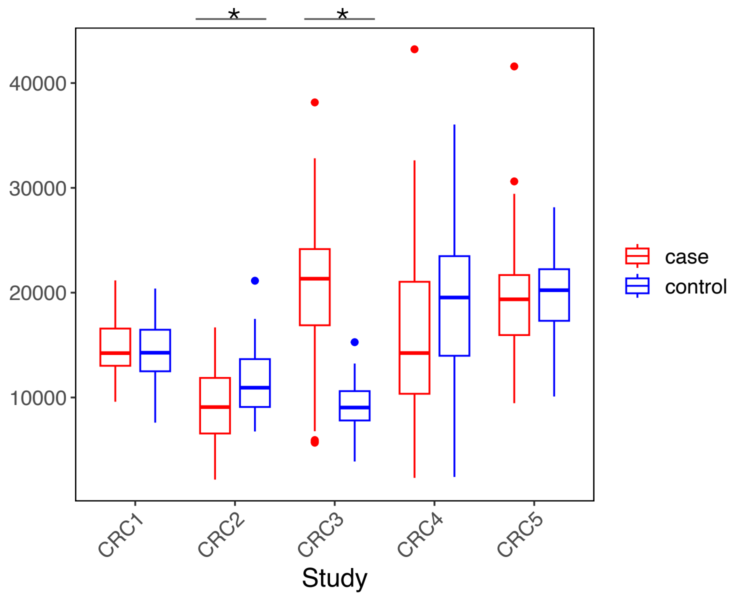

Fig. S5: Box plots of sequencing depth for cases and controls in the five metagenomics studies for colorectal cancer. The star (*) indicates the significant sequencing depth difference (Wilcoxon rank-sum test $P < 0.01$) between cases and controls. Each box indicates the median (center line), the first and third quartiles (box edges), whiskers extending to 1.5 times the interquartile range, and individual outliers (dots).

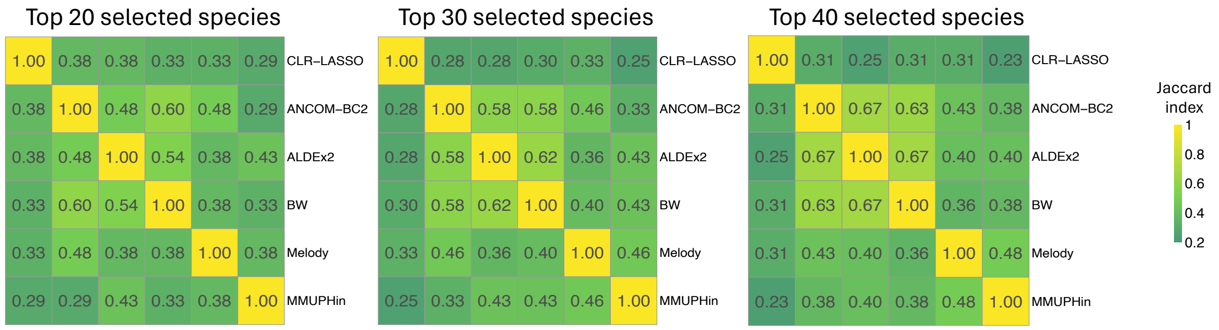

Fig. S6: Heatmap of the Jaccard index of the top N ($N = 20, 30, 40$) selected species across different methods in the meta-analysis of five metagenomics studies for colorectal cancer. The methods are displayed in the same order as in Fig. 3b. Results of all methods were generated on the original data.

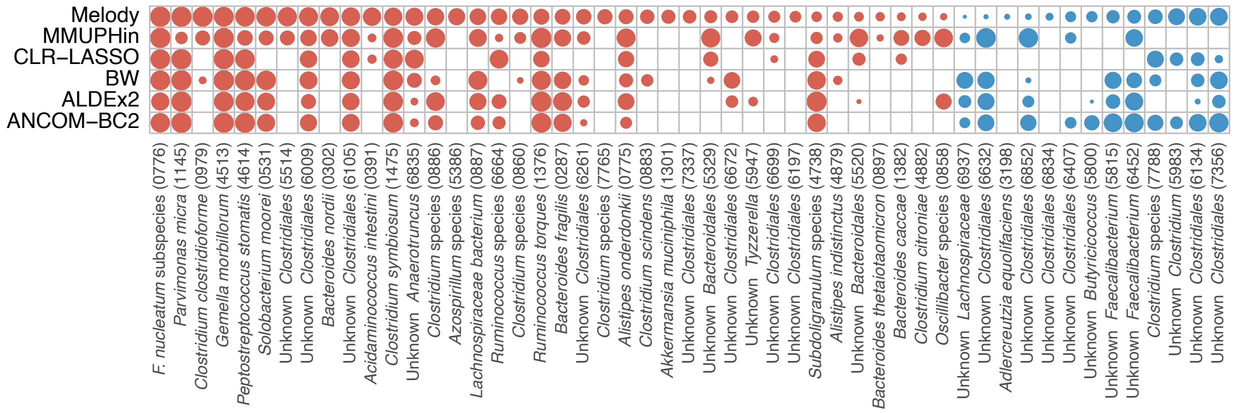

Fig. S7: Comparison of the ranking of microbial features by different methods in the meta-analysis of five metagenomics studies for colorectal cancer. The 51 Melody-identified signatures are listed at the bottom in the same order as in Fig. 3a. The circle size is proportional to the feature's ranking using the method for the row. The ranks beyond 51 are not displayed. The red color indicates positive effects, and the blue color indicates negative effects. Results of all methods were generated on the original data.

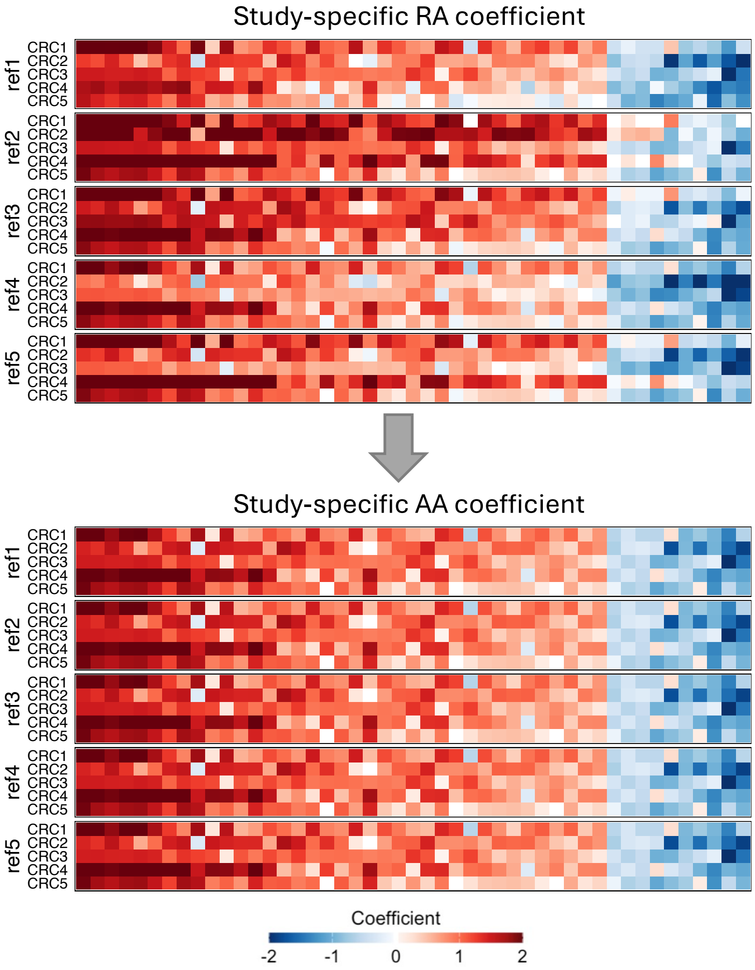

Fig. S8: Relative-abundance (RA) and absolute-abundance (AA) association coefficient estimates under different choices of reference in the Melody meta-analysis of five metagenomics studies for colorectal cancer. The upper panel shows the RA coefficient estimates under five references (the same five references in Fig. 3c). The lower panel shows the corresponding Melody-recovered AA coefficient estimates. The columns represent the shared Melody signatures identified across all five reference choices.

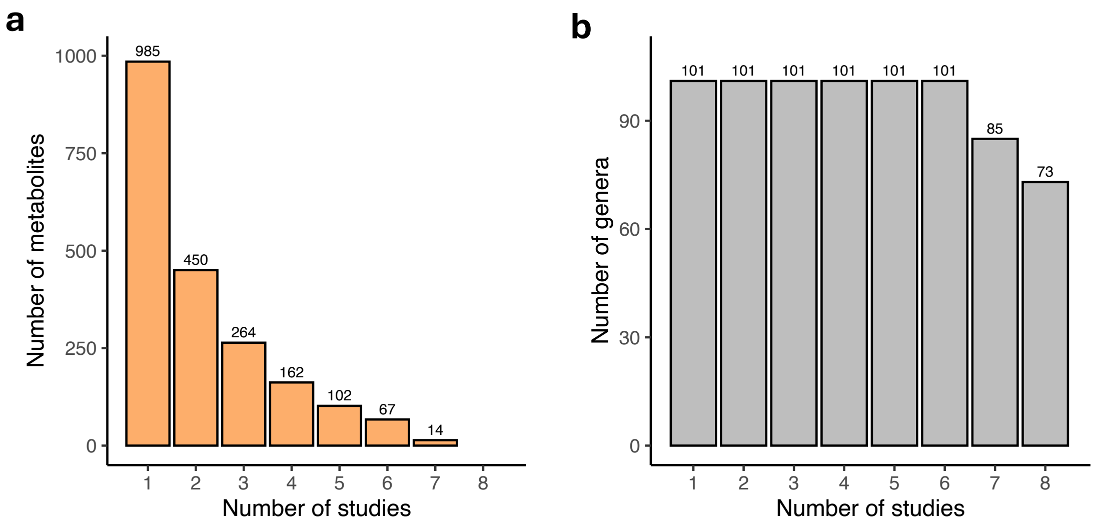

Fig. S9: Summary of the (a) metabolite and (b) genus availability across the eight microbiome-metabolome association studies. Each bar represents the number of unique metabolites (genera) that appear in at least the specified number of studies. We focused on metabolites and genera with at least 10% prevalence within individual studies.

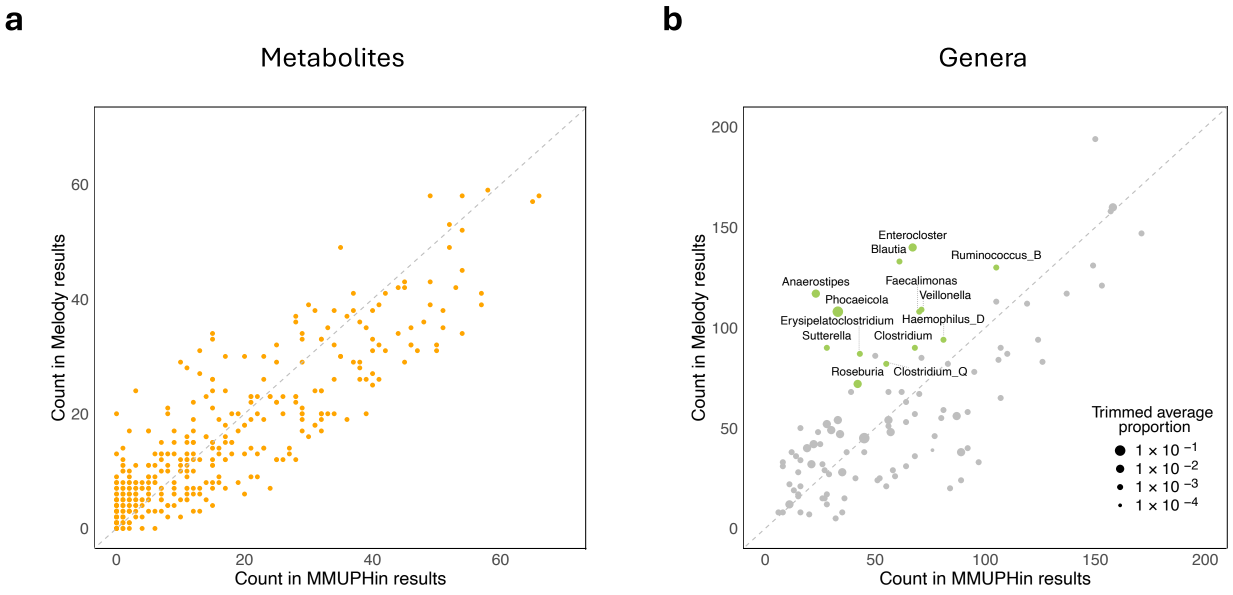

Fig. S10: Comparison of the number of selected metabolite-genus pairs by Melody and MMUPHin meta-analysis of the eight microbiome-metabolome association studies. **a**, Each dot is for one metabolite, and the count is the number of selected genera associated with the metabolite. **b**, Each dot is for one genus, and the count is the number of selected metabolites associated with the genus. The dot size is proportional to the trimmed average proportion of the genus in all samples of the eight studies. The highlighted green dots represent the group of genera that are positively associated with most of the top metabolites shown in Fig. 6.

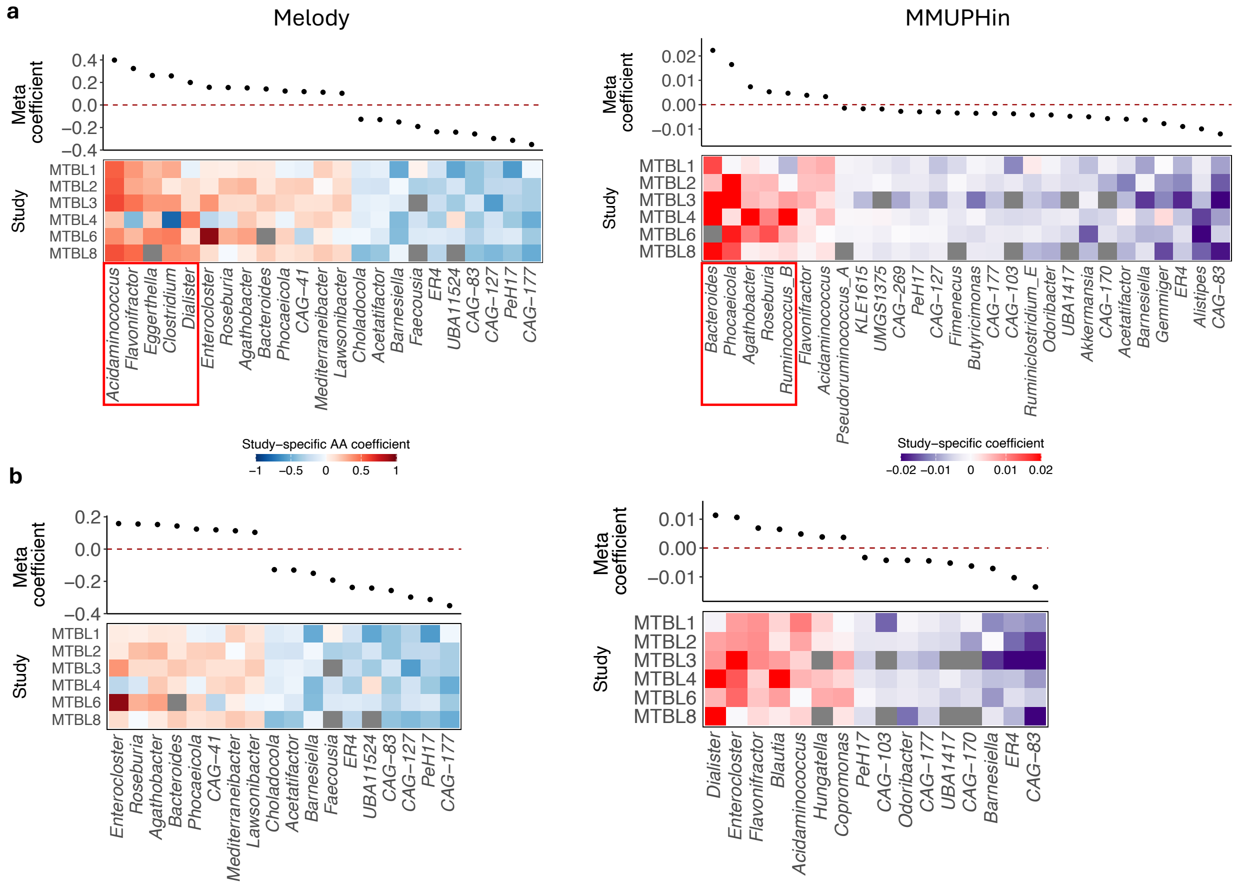

Fig. S11: Comparison of the Melody and MMUPHin signature selection stability for metabolite 4-Trimethylammoniobutanoic acid before and after genus removal. **a**, Meta association coefficients in the scatter plot and study-specific association coefficients in the heatmap for signatures identified by Melody and MMUPHin. The 5 genera that will be removed for each method are highlighted in red boxes. **b**, Meta association coefficients in the scatter plot and study-specific association coefficients in the heatmap for signatures identified by Melody and MMUPHin after genus removal. In all heatmaps, the gray cell indicates that the genus (on the column) is not available in the study (on the row).

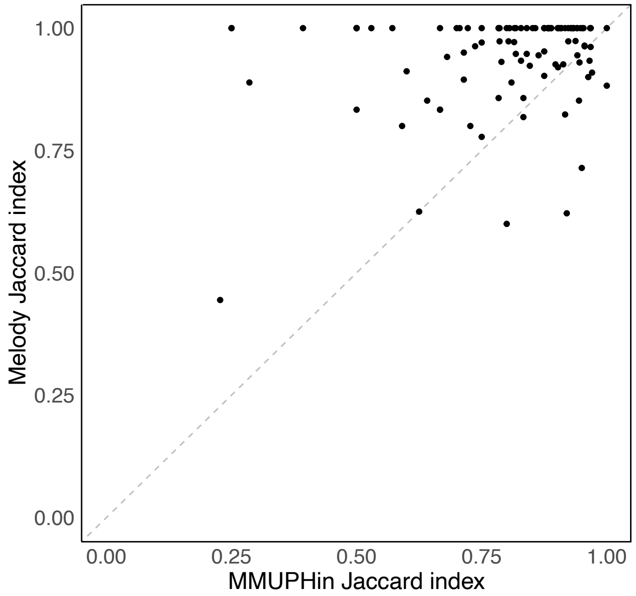

Fig. S12: Comparison of the Melody and MMUPHin signature selection stability for 106 metabolites before and after genus removal. Each dot is a metabolite that has at least 5 positively associated genera in Melody and MMUPHin results. For each method, we removed 5 genera with the highest positive meta association identified by the method and reran the meta-analysis. The Jaccard index measures the overlap of the identified signatures before and after genus removal.

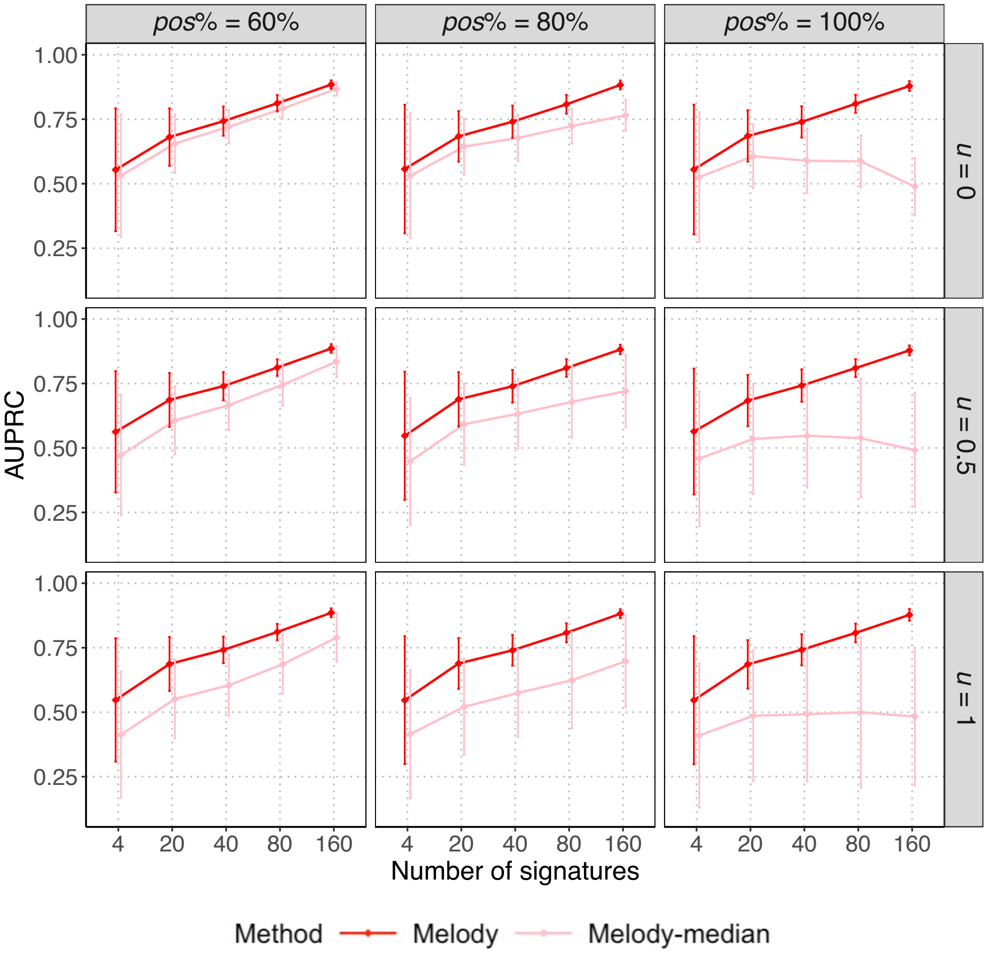

Fig. S13: Precision-recall evaluation of two Melody-related meta-analysis signature selection approaches in simulated data of five studies. Melody-median is different from Melody in that the per-study scaling factor δ_ℓ was fixed to the median of the RA coefficient estimates over features. This simulation is conducted under the same settings as in the left panel of Fig. 2. The columns of the plots represent various degrees of sign imbalance among signatures ($pos\%$ denotes the percentage of signatures with positive effects); the rows of the plots represent various degrees of sequencing depth unevenness between the two groups of interest (u denotes the sequencing depth relative change ratio for the group with higher sequencing depth, and 0 indicates no unevenness). Each panel displays the mean area under the precision-recall curve (AUPRC) with $\pm$ standard errors (indicated by error bars) based on 100 simulation replicates.

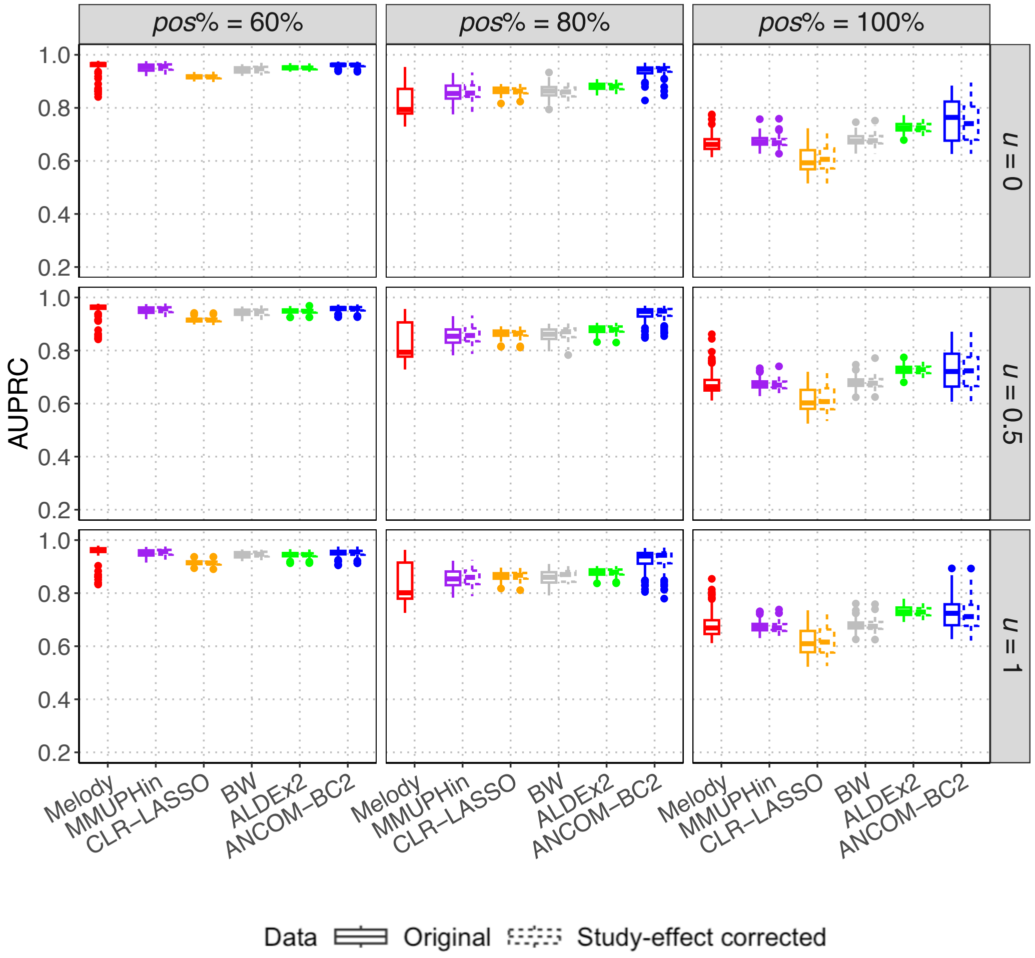

Fig. S14: Precision-recall evaluation of different meta-analysis signature selection approaches in simulated data of five studies with dense association signals. This simulation is conducted under the same settings as in the left panel of Fig. 2, except that the number of signatures is fixed at 320 (80% of the features). Melody was applied to the original data of the five studies, and the other methods were applied to both the original data and study-effect corrected data using the MMUPHin batch-effect correction algorithm. The columns of the plots represent various degrees of sign imbalance among signatures (*pos*% denotes the percentage of signatures with positive effects); the rows of the plots represent various degrees of sequencing depth unevenness between the two groups of interest (u denotes the sequencing depth relative change ratio for the group with higher sequencing depth, and 0 indicates no unevenness). Each panel displays the box plots of the area under the precision-recall curve (AUPRC) based on 100 simulation replicates. Each box indicates the median (center line), the first and third quartiles (box edges), whiskers extending to 1.5 times the interquartile range, and individual outliers (dots).

Table S1: Datasets included in the meta-analysis of microbiome association studies for colorectal cancer.

Study ID	Reference	Country	No. Case	No. Control
CRC1	Feng et al.[5]	Austria	46	63
CRC2	Yu et al.[6]	China	73	54
CRC3	Wirbel et al.[7]	Germany	60	60
CRC4	Zeller et al.[8]	France	53	61
CRC5	Vogtmann et al.[9]	United States	52	52

Table S3: Datasets included in the meta-analysis of microbiome-metabolome association studies.

Study ID	Reference	Correlated Sample Y/N	No. Subjects Control/Case	No. Samples Control/Case	Microbiome 16S/WGS	Metabolome Target/Untargeted
MTBL1	Erawijantari et al.[10]	N	54/42	54/42	WGS	Targeted
MTBL2	Yachida et al.[11]	N	74*/220	74*/220	WGS	Targeted
MTBL3	Kim et al.[12]	N	102/138	102/138	16S	Untargeted
MTBL4	Franzosa et al.[13]	N	56/164	56/164	WGS	Untargeted
MTBL5	Mars et al.[14]	Y	24/51	139/305	WGS	Targeted
MTBL6	Lloyd-Price et al.[15]	Y	26/79	104/278	WGS	Untargeted
MTBL7	Wang et al.[16]	N	67/220	67/220	WGS	Untargeted
MTBL8	Poyet et al.[17]	Y	83/NA	164/NA	16S	Untargeted

*: the number is after removing control subjects shared with MTBL1.

Tables S2, S4, S5 are in Additional file 2.

References

1. Zhu J, Wang X, Hu L, Huang J, Jiang K, Zhang Y, et al. abess: a fast best-subset selection library in python and R. The Journal of Machine Learning Research. 2022;23(1):9206–9212.

2. Powell MJ. An efficient method for finding the minimum of a function of several variables without calculating derivatives. The Computer Journal. 1964;7(2):155–162.

3. Heath MT. Scientific computing: an introductory survey, revised second edition. SIAM; 2018.

4. Kiefer J. Sequential minimax search for a maximum. Proceedings of the American Mathematical Society. 1953;4(3):502–506.

5. Feng Q, Liang S, Jia H, Stadlmayr A, Tang L, Lan Z, et al. Gut microbiome development along the colorectal adenoma–carcinoma sequence. Nature Communications. 2015;6(1):6528.

6. Yu J, Feng Q, Wong SH, Zhang D, yi Liang Q, Qin Y, et al. Metagenomic analysis of faecal microbiome as a tool towards targeted non-invasive biomarkers for colorectal cancer. Gut. 2017;66(1):70–78.

7. Wirbel J, Pyl PT, Kartal E, Zych K, Kashani A, Milanese A, et al. Meta-analysis of fecal metagenomes reveals global microbial signatures that are specific for colorectal cancer. Nature Medicine. 2019;25:679–689.

8. Zeller G, Tap J, Voigt AY, Sunagawa S, Kultima JR, Costea PI, et al. Potential of fecal microbiota for early-stage detection of colorectal cancer. Molecular Systems Biology. 2014;10(11):766.

9. Vogtmann E, Hua X, Zeller G, Sunagawa S, Voigt AY, Hercog R, et al. Colorectal cancer and the human gut microbiome: reproducibility with whole-genome shotgun sequencing. PloS One. 2016;11(5):e0155362.

10. Erawijantari PP, Mizutani S, Shiroma H, Shiba S, Nakajima T, Sakamoto T, et al. Influence of gastrectomy for gastric cancer treatment on faecal microbiome and metabolome profiles. Gut. 2020;69(8):1404–1415.

11. Yachida S, Mizutani S, Shiroma H, Shiba S, Nakajima T, Sakamoto T, et al. Metagenomic and metabolomic analyses reveal distinct stage-specific phenotypes of the gut microbiota in colorectal cancer. Nature Medicine. 2019;25(6):968–976.

12. Kim M, Vogtmann E, Ahlquist DA, Devens ME, Kisiel JB, Taylor WR, et al. Fecal metabolomic signatures in colorectal adenoma patients are associated with gut microbiota and early events of colorectal cancer pathogenesis. MBio. 2020;11(1):10–1128.

13. Franzosa EA, Sirota-Madi A, Avila-Pacheco J, Fornelos N, Haiser HJ, Reinker S, et al. Gut microbiome structure and metabolic activity in inflammatory bowel disease. Nature Microbiology. 2019;4(2):293–305.

14. Mars RA, Yang Y, Ward T, Houtti M, Priya S, Lekatz HR, et al. Longitudinal multi-omics reveals subset-specific mechanisms underlying irritable bowel syndrome. Cell. 2020;182(6):1460–1473.

15. Lloyd-Price J, Arze C, Ananthakrishnan AN, Schirmer M, Avila-Pacheco J, Poon TW, et al. Multi-omics of the gut microbial ecosystem in inflammatory bowel diseases. Nature. 2019;569(7758):655–662.

16. Wang X, Yang S, Li S, Zhao L, Hao Y, Qin J, et al. Aberrant gut microbiota alters host metabolome and impacts renal failure in humans and rodents. Gut. 2020;69(12):2131–2142.

17. Poyet M, Groussin M, Gibbons SM, Avila-Pacheco J, Jiang X, Kearney SM, et al. A library of human gut bacterial isolates paired with longitudinal multiomics data enables mechanistic microbiome research. Nature Medicine. 2019;25(9):1442–1452.
